# What Resources Do NHS Commissioning Organisations Use to Support Antimicrobial Stewardship in Primary Care in England?

**DOI:** 10.3390/antibiotics9040158

**Published:** 2020-04-02

**Authors:** Rosalie Allison, Donna M. Lecky, Elizabeth Beech, Diane Ashiru-Oredope, Céire Costelloe, Rebecca Owens, Cliodna A.M. McNulty

**Affiliations:** 1 Public Health England, Gloucester GL1 3NN, UK; Donna.Lecky@PHE.gov.uk (D.M.L.); Diane.Ashiru-Oredope@phe.gov.uk (D.A.-O.); rebecca.owens@open.ac.uk (R.O.);; 2 NHS Improvement, London BA2 5RP, UK; elizabeth.beech@nhs.net; 3 Global Digital Health Unit, Department of Primary Care and Public Health, Imperial College London, London W6 8RP, UK; ceire.costelloe@imperial.ac.uk

**Keywords:** health education, training, TARGET, Antibiotic Guardian, antimicrobial resistance

## Abstract

Professional education and public engagement are fundamental components of any antimicrobial stewardship (AMS) strategy. The National Institute for Health and Care Excellence (NICE), Public Health England (PHE), Health Education England (HEE) and other professional organisations, develop and publish resources to support AMS activity in primary care settings. The aim of this study was to explore the adoption and use of education/training and supporting AMS resources within NHS primary care in England. Questionnaires were sent to the medicines management teams of all 209 Clinical Commissioning Groups (CCGs) in England, in 2017. Primary care practitioners in 168/175 (96%) CCGs received AMS education in the last two years. Respondents in 184/186 (99%) CCGs reported actively promoting the TARGET Toolkit to their primary care practitioners; although 137/176 (78%) did not know what percentage of primary care practitioners used the TARGET toolkit. All respondents were aware of Antibiotic Guardian and 132/167 (79%) reported promoting the campaign. Promotion of AMS resources to general practices is currently excellent, but as evaluation of uptake or effect is poor, this should be encouraged by resource providers and through quality improvement programmes. Trainers should be encouraged to promote and highlight the importance of action planning within their AMS training. AMS resources, such as leaflets and education, should be promoted across the whole health economy, including Out of Hours and care homes. Primary care practitioners should continue to be encouraged to display a signed Antibiotic Guardian poster as well as general AMS posters and videos in practice, as patients find them useful and noticeable.

## 1. Introduction

Antibiotic prescribing in primary care has been shown to directly affect antimicrobial resistance (AMR) [1]; in 2017, 81% of prescribing occurred in primary care, in England; 23% of which were deemed inappropriate, often for non-bacterial infections such as upper respiratory tract infections [1,2,3]. In the European Union (EU) alone, 25,000 deaths annually are attributed to antibiotic resistance, with extra healthcare costs and lost productivity of at least €1500 million [4].

The UK’s five year national action plan for tackling AMR (2019–2024) sets out actions to address the key challenges with the overarching goal to effectively contain and control resistance [5]. These include improving professional education, training and public engagement, including education on evidence-based antimicrobial prescribing guidance [6] and consultation communication skills—fundamental components of any antimicrobial stewardship (AMS) strategy [5,7]. Professional education in England is overseen by Health Education England (HEE) and is supported by free and openly available toolkits and campaigns, such as TARGET (Treat Antibiotics Responsibly, Guidance, Education, Tools) [8], Antibiotic Guardian [9], European Antibiotic Awareness Day (EAAD) [10] and World Antibiotic Awareness Week (WAAW) [11]. 

### 1.1. TARGET (Treat Antibiotics Responsibly, Guidance, Education, Tools)

TARGET, launched by Public Health England (PHE) and the Royal College of General Practitioners (RCGP) (2012), aims to support appropriate antimicrobial prescribing in primary care [8]. Toolkit resources include patient facing information leaflets (including information on expected duration of infection, self-care and back-up prescriptions); clinical and waiting area resources (such as videos and posters); educational training resources (presentations to be used face-to-face with primary care practitioners; e-learning infection related clinical courses); audit toolkits; quick reference diagnostic tools; and self-assessment checklists for antimicrobial stewardship. 

### 1.2. Antibiotic Guardian Campaign

Antibiotic Guardian, developed by PHE (2014), aims to improve knowledge and behaviours regarding antibiotic prescribing and use among both healthcare professionals and the public through an online action-based pledge system [9]. Although used as part of UK activities for EAAD, the campaign is available all year round, in support of the UK five-year AMR action plan [5]. 

Apart from anecdotal feedback, there has been no comprehensive study of the use of either of these resources in the English primary care setting.

### 1.3. Aim

To explore the adoption and use of education/training and the widely available PHE AMS resources within NHS primary care in England.

## 2. Results

Overall, AMS leads representing 187/209 (89%) Clinical Commissioning Groups (CCGs) (clinically-led statutory NHS bodies responsible for the planning and commissioning of health care services for their local area) responded to the questionnaire: 175/187 (94%) to the AMS education/training section; 186/187 (99%) to the TARGET section; 167/187 (89%) to the Antibiotic Guardian section. The AMS leads were generally pharmacists in roles such as prescribing advisers. Not all respondents answered all questions; therefore, results are represented as a percentage of CCGs that responded to each question. See Supplementary File S1 for a full breakdown of responses.

### 2.1. AMS Education/Training

Respondents reported that primary care practitioners in 168/175 (96%) responding CCGs had received AMS education/training in the last two years (2015-17). Of these, 140/168 (83%) CCGs stated that the practitioners had received face-to-face education/training and 121/168 (72%) had signposted/referred practitioners to e-learning; 94/168 (56%) had invited practitioners to participate in both. Staff to whom AMS education/training was focused included:

• GP staff (165/166; 99% responding CCGs)
  o Antimicrobial prescribers (112/166; 67%)
  o All GP staff (69/166; 42%)
• Out of hours (OOH) staff (46/166 28%;)
• Care home staff (41/166; 25%)
• Community pharmacists (30/166; 18%)

Respondents also commented that the focus had also been the GP prescribing lead (*n* = 4); community nursing teams (*n* = 10); dentists (*n* = 4); and public/patients (*n* = 3). 

### 2.2. Face-to-Face AMS Education/Training

Face-to-face training was delivered by pharmacists in 112/129 (87%) responding CCGs; 69/129 (53%) by the AMS lead within the Medicines Management Team or CCG prescribing advisor (67/129; 52%). Microbiologists were involved in training in 76/129 (59%) responding CCGs. MMTs representing five CCGs commented that infection prevention and control nurses were also involved. 

Participants in 117/132 (89%) responding CCGs included antimicrobial prescribing data in local AMS education/training; only 21/132 (16%) reported including detailed action planning. Respondents in 83/132 (63%) CCGs reported developing their own, local, AMS education/training, using: TARGET resources (*n* = 27), local guidelines (*n* = 17), national guidelines (*n* = 14), including NICE (*n* = 6) and PHE guidelines (*n* = 6), North of England Commissioning Support (NECS) AMS programme (*n* = 8), learning from other practices/CCGs (*n* = 5). See  Supplementary File S1 for a full breakdown of responses.

Of the 106/128 (83%) responding CCGs that knew the percentage of primary care practices that had received face-to-face AMS training in the previous two years (2015-17), 73/106 (69%) CCGs reported >75% of their primary care practices having received face-to-face AMS education/training during this time.

### 2.3. AMS E-learning

Of those that reported signposting to AMS e-learning, 117/119 (98%) reported signposting their primary care practitioners to TARGET eLearning resources; 33/119 (28%) to HEE and e-LfH (eLearning for Healthcare) Level 1 AMR; 20/119 (17%) to NICE AMS course; 18/119 (15%) to CPPE (Centre for Pharmacy Postgraduate Education) resources; 10/119 (8%) to the British Society for Antimicrobial Chemotherapy’s MOOC (Massive Open Online Course). Eight CCGs indicated ‘other’: five of which mentioned signposting to the NECS (North of England Commissioning Support) e-learning module.

Of those that reported signposting to TARGET e-learning: 77/103 (75%) reported signposting their primary care practitioners to the TARGET antibiotic webinar series13; 73/103 (71%) to the TARGET Antibiotic Resistance in Primary Care e-module14; 47/103 (46%) to the UTI e-learning15; 31/103 (30%) to MARTI Managing Acute Respiratory Tract Infections; 20/103 (19%) to STAR: Stemming the Tide of Antibiotic Resistance e-module16; 19/103 (18%) to Skin Infections online course17; 13/103 (13%) to Sexual Health in Primary Care18; 11/103 (11%) to Managing infectious diarrhoea19; 9/103 (9%) to National Prescribing Centre (NPC) e-learning.

Positive comments about AMS education/training included providing ideas that could be implemented locally (*n* = 19), such as an opportunity for peer learning (*n* = 4), enabling clinicians to action plan (*n* = 3), providing practical tips on appropriate prescribing (*n* = 2), helping to reduce workload (*n* = 2).

Nineteen CCGs commented that AMS education/training gave primary care practitioners the opportunity to refresh on current guidance and evidence (*n* = 19), helped raise awareness (*n* = 18); gave the opportunity to review current practice (*n* = 12), and challenged preconceived notions that may impact negatively on practice (*n* = 3). Furthermore, it helped prioritise and focus on AMS (*n* = 6).

*“I feel the education sessions are usually thought provoking and force you to reassess your approach to managing AMS whether it be by changes to prescribing or raising awareness.” (CCG-67—Questionnaire)*

Participants commented that the ability to access e-learning at any point was the major advantage (*n* = 3). Linking the e-learning to Continuing Professional Development (CPD) was also a facilitator for completion of a module (*n* = 2). Issues identified with AMS education/training included: time (*n* = 11), reaching the correct people (*n* = 10), unsure which e-learning to promote (*n* = 5) (Box 1).

Box 1 **Facilitators, suggestions and barriers of AMS education/training, reported by Medicines Management AMS leads.**

**Facilitators or suggestions for AMS education/training**Link to local schemes (*n* = 18), e.g., adding to prescribing incentive/engagement scheme (*n* = 10), including in primary care commissioning framework (*n* = 1), GP quality contract (*n* = 1)“GPs will undertake training readily if
incentivised or it greatly reduces their workload or enable protected time.” (CCG-153—Questionnaire)Training delivered by key staff (*n* = 16).
a.Specific people are facilitators of training, e.g., practice pharmacist, microbiologists (*n* = 6)
*“Training needs to be delivered by someone senior to them, i.e., a microbiologist.” (CSU-6; CSU-13; CSU-17—Questionnaire)*
b.GPs appreciated opportunity to directly question the consultant (*n* = 4)c.Useful to have an expert who understands the evidence and also the perspective of the busy GP and primary care (*n* = 2)“I think this is useful especially when delivered by recognised peers such as our local microbiologist whose husbandincidentally is a local GP.” (CCG-115—Questionnaire)Important to focus on whole practice/system-wide approach (*n* = 11).“Ideally this should be a widespread and wholly integrated approach—primary and secondary care—supported by PHE.” (CCG-117—Questionnaire)Short, snappy, focused, engaging (*n* = 9).“[In reference to e-learning] Need to be more interactive and updated to keep GPs interested and willing to ’repeat’ the same training.” (CCG-11—Questionnaire)Link to national schemes (*n* = 7), e.g., CPD points (*n* = 1), AMS education, training for student clinicians (nurses, GPs, etc.) (*n* = 2), could training be part of the GP Deanery (regional organisation responsible for postgraduate medical training) (*n* = 1), Include in GP appraisal process (*n* = 2), Mandatory AMR training module as part of revalidation (*n* = 1), link to QP (*n* = 1)**Barriers of AMS education/training**Time (*n* = 11)“GPs very busy and don’t always make time to do training even if highlighted as useful.” (CCG-103—Questionnaire)“Local microbiologist supportive however trying to gain times which are appropriate for all is difficult.” (CSU-6; CSU-13; CSU-17—Questionnaire)Reaching the correct people (*n* = 10)“Clinicians who attend and take part in training tend to be the engaged clinicians who are aware of the AMS agenda and prescribe carefully. It’s often the less engaged clinicians who don’t attend training we need to educate more about AMS and change their antibiotic prescribing behaviours.” (CSU-1; CSU-8; CSU-11—Questionnaire)Unsure of which e-learning to promote (*n* = 5)“Excellent resources but there are a wide array to access... could be confusing which are the best/most effective useof limited time to undertake?” (Other-8—Questionnaire)

Participants in 100/117 (85%) responding CCGs reported that they did not monitor how many primary care practitioners had completed the e-learning that they signpost to. For the few that did monitor, this was reported by asking practitioners to provide evidence of completion (*n* = 10), either as part of their incentive scheme, often linked to the QP targets (*n* = 5), or as part of their primary care antimicrobial work programme (*n* = 2). MMTs representing two CCGs reported that NECS (North of England Commissioning Support) provided these data as they developed the e-learning they signpost to.

### 2.4. TARGET

Fifty-four CCGs gave positive free-text feedback about the TARGET toolkit, overall.

*“Excellent resource overall—trusted, accessible, comprehensive.” (Other-8—Questionnaire)*

The difficulty mentioned most frequently was around time to maximise use, both for practitioners to access the TARGET resources, and also to implement them in everyday practice (*n* = 8).

*“The resources are great… The issue is about clinicians having the time to digest the information and motivation to utilise it in clinical settings.” (CCG-17; CCG-40; CCG-102; CCG-104—Questionnaire)*

Monitoring usage and measuring success of TARGET resources in supporting AMS in primary care.

A total of 184/186 (99%) responding CCGs reported actively promoting the TARGET toolkit to their primary care practitioners; although 137/176 (78%) reported that they did not know what percentage of primary care practitioners used it. Three CCGs mentioned using incentive schemes or feedback from practice Medicines Management Teams to monitor uptake.

MMTs commented that they promote the toolkit, but do not have the resource to monitor use or evaluate success of the resources in supporting AMS.

#### 2.4.1. Leaflets to Share with Patients

**Figure 1:**
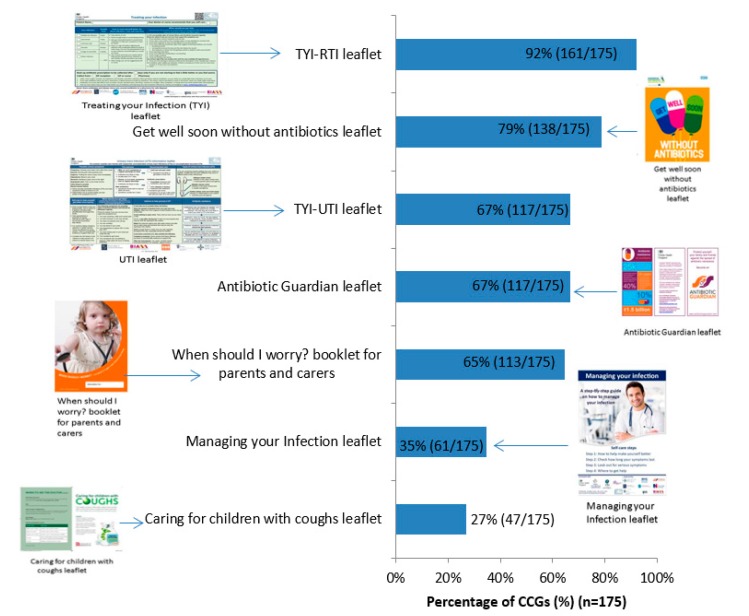
Percentage of Clinical Commissioning Groups (CCGs) that have actively promoted each leaflet to share with patients, hosted on the TARGET website (*n* = 175) (4 indicated none, 7 don’t know, 1 blank = 187 CCGs total).

Between 47 and 161 (27–92%) responding CCGs reported actively promoting each of the different leaflets to share with patients, hosted on the TARGET website. The Treating Your Infection Respiratory Tract Infection (TYI-RTI) leaflet was reported to be the most widely promoted by 161/175 (92%) responding CCGs (Figure 1). 

**Figure 2:**
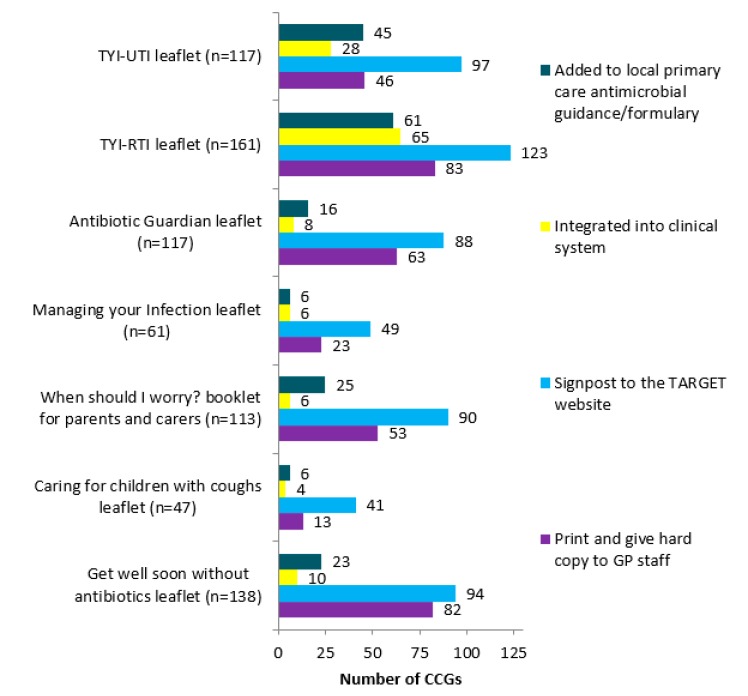
Breakdown of means of actively promoting the leaflets to share with patients, hosted on the TARGET website.

The patient leaflets were usually signposted to on the TARGET website (Figure 2) or printed by MMTs and hard copies given to GP staff. Respondents in 65/157 (41%) CCGs reported integrating the TYI-RTI leaflet into GP clinical practice. In a free-text comments box, some MMTs reported emailing the practitioners (*n* = 7), including in newsletter (*n* = 7), promoting at locality meetings (*n* = 5), and tweeting (*n* = 4).

**Figure 3:**
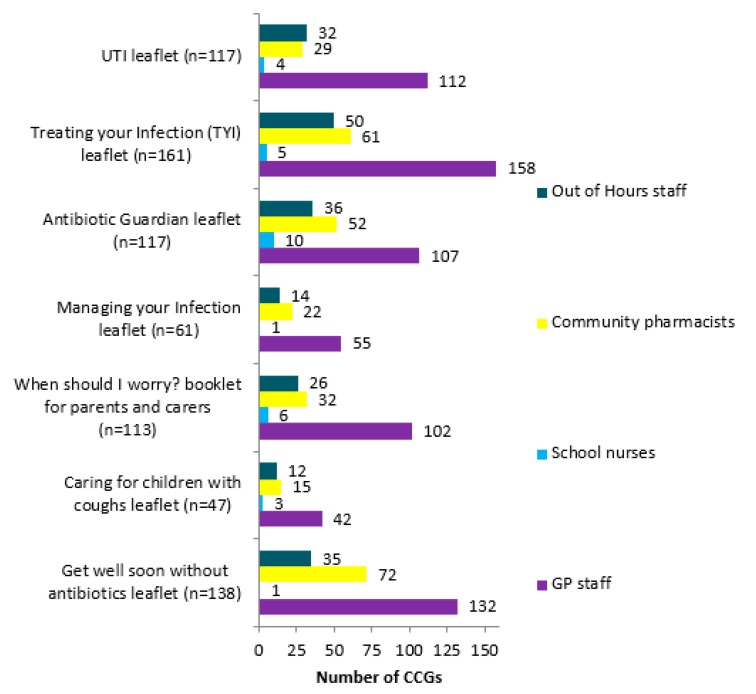
Breakdown of who the TARGET leaflets are reportedly promoted to.

GP staff (Figure 3) were the main focus for TARGET leaflets promotion. Other promotional routes included care home staff (*n* = 6), community services (*n* = 4), community matrons (*n* = 6), community nurses (*n* = 9). MMTs representing 21 CCGs commented that they had received positive feedback from practitioners about the leaflets, including that practitioners found them useful for patient education (*n* = 15), GP decision making (*n* = 7), helping with patient’s satisfaction of consultation (*n* = 2).

*“GPs report that they find the leaflets helpful. It backs up what they tell the patient or their carer and gives them the safety netting advice.” (CCG-96—Questionnaire)*

Lack of funding to print hard copies was reported as the main barrier to use (*n* = 7); therefore, staff needed to find alternative electronic means of promotion. 

*“Funding for printing and distribution is a problem, plus resource to manage requests for printed documents.” (CSU-6; CSU-13; CSU-17—Questionnaire)*

#### 2.4.2. Resources for Clinical and Waiting Areas

A total of 146/186 (78%) responding CCGs reported actively promoting resources for clinical and waiting areas, hosted on the TARGET website. Posters (137/146; 94% of responding CCGs) were the most commonly promoted, followed by videos (84/146; 58%) and Self Care Forum fact sheets (55/146; 38%).

Although patient education was mentioned by six respondents as a positive impact of the resources for clinical and waiting areas; eight questioned the impact, especially in relation to the positioning of posters. 

*“GP surgeries have to put millions of posters up, [they] never refresh [them]. [We] don’t know how successful each is; GP surgeries don’t make best use of resources.” (CCG-16; CCG-38; CCG-98—Questionnaire)*

Other issues mentioned, include: problems with the videos (*n* = 8), for example, TV is sponsored, formatting, cannot have audio, do not have video capability; rumour that it is a Care Quality Commission (CQC—The independent regulator of health and social care in England) requirement for laminated posters in consultation rooms due to infection prevention controls (*n* = 4).

#### 2.4.3. Self-assessment Checklist (SAC) for Practitioners

Over half of responding CCGs (98/180; 54%) reported actively promoting the TARGET SAC for practitioners in their locality. The checklist helps clinicians to assess their current AMS against others, regionally and nationally, and links to action planning, suggesting approaches to improve stewardship activities.

However, MMTs representing seven CCGs reported that practitioners often did not use the SAC, suggesting that this may be due to lack of incentive (*n* = 1) or awareness of the checklist (*n* = 1). MMTs representing 10 CCGs mentioned incentivising completion of the SAC, and it was also reported suggested that inclusion of AMS in CQC inspections had raised its profile. 

“In 2014/15 in ** we asked all GP practices to complete the checklist as part of a review for the incentive scheme. They were asked to do it twice—once before the end of Oct with the aim of implementing any recommendations and then again before the end of March to see what improvements had been made.” (CCG-15—Questionnaire)

### 2.5. Antibiotic Guardian Campaign

All 167 responding CCGs reported that they had heard of the Antibiotic Guardian Campaign and 132/167 (79%) reported actively encouraging primary care practitioners (129/132 (98%)) or patients and the public (74/132 (56%)) to become Antibiotic Guardians.

Free-text responses indicated that promotion of Antibiotic Guardian to primary care practitioners was mainly attained by direct messaging (*n* = 99), for example, face-to-face (*n* = 34), newsletters (*n* = 31), email and email signature (*n* = 26), or letters to practices (*n* = 6). In contrast, indirect messaging (*n* = 40), for example, social media (*n* = 19), CCG website (*n* = 14), local newspapers (*n* = 7) were most utilised to encourage patients and the public to become antibiotic guardians. Patient facing materials, for example, posters, leaflets, videos, badges were used for both groups, as well as linking to WAAW and EAAD.

Nine MMTs commented that the AG campaign helped raise awareness.

*“Raises awareness by key messages in target areas, for example, waiting rooms, community pharmacies.” (CCG-68—Questionnaire)*

Reported issues, mentioned, include no follow up (*n* = 17), unsure whether Antibiotic Guardian changes attitudes or behaviours (*n* = 13), lack of prescriber engagement (*n* = 11), lack of public focus and engagement (*n* = 6).

*“Not widely understood, especially by patients even after explanation. Hesitance by patients to sign up as they don’t know what they will have to do moving forwards. Signing up does not result in anything—needs following up with patient focussed information to public who have signed up.” (CCG-119—Questionnaire)*

MMTs representing four CCGs suggested that Antibiotic Guardian needs a new hook, such as case studies or a higher profile.

*“I think it may need case studies to promote problems to public as to what happens to that person might happen to them—shock tactics.” (CCG-78—Questionnaire)*

## 3. Discussion

Provision and promotion of AMS education and training was undertaken by virtually all CCGs within the last two years, in the form of both face-to-face and e-learning; only 21/132 (16%) responding CCGs reported including detailed action planning in their face-to-face AMS education/training. Action planning and communicating these recommendations in an educational manner aids acceptance of the recommendations, and therefore should be addressed for future delivery [12]. TARGET is the most signposted AMS e-learning provider, with the TARGET webinar series being the most widely promoted. The general TARGET AMR in Primary care e-module is more popular than the individual conditions’ modules, and therefore, should be maintained. Time to attend education/training is one of the main barriers for primary care practitioners, whilst e-learning has the flexibility of access at any point. Taking this time pressure into account, ideas to facilitate future up-take, based on participants’ comments, include screen-casting webinars and shorter educational workshops/e-learning. From literature, other strategies to increase attendance at educational events include: using mixed interactive and didactic formats; and focusing on outcomes that are likely to be perceived as serious [13].

A survey of UK postgraduate medical training has highlighted that coverage of antimicrobial resistance and stewardship related topics and learning points are poor [14] and studies show that training practices, on average, prescribe fewer antimicrobials and a smaller proportion of broad spectrum antibiotics than non-training practices, highlighting the importance of education and training in appropriate prescribing [15]. Recent research highlights that TARGET, as a whole, is useful for supporting AMS in primary care [16] and leads to more appropriate antibiotic prescribing [17]. As such, postgraduate medical training providers should include AMR and AMS learning points to instil appropriate prescribing early on in prescribers’ careers, utilising the existing TARGET resources. Additionally, qualitative research has also found that GP staff find the TARGET patient information leaflets the most useful resource, especially if made accessible through computer shortcuts built into general practice software [16]. This study found that many CCGs had integrated the TARGET leaflets into their clinical systems to increase accessibility and reduce printing costs, which should be encouraged. 

An evaluation of Antibiotic Guardian found that it effectively raised awareness of antimicrobial resistance/stewardship [18] and so should continue to be widely promoted. AMS leads reported that some GP practices display signed posters of their prescribers’ Antibiotic Guardian pledges for patients to see. A personalised public commitment poster for AMS results in more appropriate antibiotic prescribing in American University primary care clinics [19]. This public display of AMS pledges should be encouraged as it may increase opportunity and motivation of staff to consider their prescribing practice and increase awareness and understanding for the general public. Some AMS leads commented that their primary care practitioners had reported issues with posters as they had been told that it was a CQC requirement to have laminated posters. The key lines of enquiry that CQC inspect against include: 

*“S1.8 How are standards of cleanliness and hygiene maintained? Are there reliable systems in place to prevent and protect people from a healthcare-associated infection? S1.9 Do the design, maintenance and use of facilities and premises keep people safe?” [20,21]*.

Inspectors do not stipulate how this is done, so long as they are assured that it is safe. The reported high percentage of CCGs promoting the use of posters and videos, in the present study, is promising and should be continued to be promoted as studies suggests that patients find them useful and noticeable [22]. Practices could be advised on poster placement or rotation to avoid patient and staff apathy.

Most MMTs in the present study reported that they do not monitor the uptake of promoted tools, or evaluate success in their use towards reducing inappropriate prescribing. Monitoring could be facilitated through local incentive/engagement schemes, which over 70% of CCGs reported having [23], and use of a SAC [24]. 

### Strengths and Limitations

This is the first study of its kind to comprehensively assess use and opinions of multiple national AMS resources and campaigns. Whereas other studies have asked small numbers of CCG staff, this questionnaire had responses from 89% of all CCGs, in 2017.

It was the MMT AMS lead that was asked to complete the questionnaire, not primary care practitioners, and therefore, uptake of resources and campaigns could not be assessed. This study provides descriptive data and does not analyse responses in relation to known prescribing data. This has highlighted a gap in monitoring and evaluation that should be addressed to provide the evidence-base for future policy recommendations.

Free text responses could be considered a deterrent as they take longer to complete than tick boxes. However, as they were optional, the inclusion of free-text responses is considered a further strength of the methodology, as participants took the opportunity to explain their answers, which gave a greater insight into real-life implementation of initiatives. 

## 4. Materials and Methods 

In 2017, AMS leads for the MMTs of all 209 CCGs, were invited, by email, to complete an online questionnaire exploring AMS activities in the CCGs they commission. 

Questions exploring implementation of resources and campaigns to support AMS covered: AMS education/training; TARGET; Antibiotic Guardian Campaign. See Supplementary File S2 for the full AMS implementation questionnaire.

Questionnaire data were exported to Excel, anonymised and descriptively analysed. Free text responses to the questionnaire were exported to NVivo and analysed by two researchers.

The participant selection and survey distribution have been described in full elsewhere [25]. 

## 5. Conclusions

### 5.1. Implications for the Development of Resources to Support AMS Activity in Primary Care Settings

To increase accessibility, reduce printing costs, and facilitate clinical audits, resources should be integrated into clinical systems and assigned Read Codes/SNOMED CT codes (clinical terminology system used in General Practice in the UK to capture symptoms, diagnosis, treatment and other criteria). Resources to support face-to-face AMS education/training should be promoted more widely, including to both local microbiologists and MMTs as they were reported as delivering the majority of face-to-face AMS education/training in primary care. 

### 5.2. Implications for NHS Commissioning Organisations

As the TARGET antibiotic workshops improve appropriate antibiotic use [17], and 99% of responding CCGs reported actively promoting TARGET, AMS strategies should continue to promote and provide AMS education to change existing behaviour. As only one quarter of CCGs currently reported including action planning in their face-to-face AMS education/training, trainers should be encouraged to promote and highlight the importance of action planning within their training sessions to facilitate implementation of suggested changes and consequently change behaviour and reduce inappropriate prescribing. The TARGET SAC for prescribers could be used to facilitate this action planning as it suggests best practice [24]. Resources to support AMS should be promoted across the whole health economy, for example, community pharmacists, OOH staff, school nurses, care homes, as AMS leads reported mainly promoting TARGET leaflets and education to GP staff. Education across healthcare is important as OOHs and care homes are often reported as areas of greater prescribing [3,26], yet respondents in the present study reported that they were not supported as well as other groups. Monitoring uptake of initiatives could be facilitated through local engagement schemes, which over 70% of CCGs reported having [23], as practitioners have to provide evidence of how they meet the requirements of the scheme, such as attending AMS education/training.

### 5.3. Implications for Primary Care Practitioners

As other research shows that displaying signed posters of their prescribers’ Antibiotic Guardian pledges for patients to see could result in more appropriate antibiotic prescribing [19], and primary care practitioners should be encouraged to display a signed Antibiotic Guardian poster in their practice. Similarly, general AMS posters and videos should continue to be displayed in practice, as patients find them useful and noticeable [22]. The location of the posters could be rotated regularly to avoid patient and staff apathy. Primary care practitioners should be encouraged to give feedback of evidence of their AMS education, audit and use of other resources, as currently, evaluation is poor. This could be facilitated through engagement schemes, Continuing Professional development (CPD) records and medicines managers.

## Supplementary Materials

The following are available online at https://www.mdpi.com/2079-6382/9/4/158/s1, File S1: Breakdown of participants responses to each question, including ‘blanks’ and ‘don’t know’, File S2: Full AMS implementation questionnaire.
