# Supplementary material for: What Resources Do NHS Commissioning Organisations Use to Support Antimicrobial Stewardship in Primary Care in England?
Source: Antibiotics (Basel). 2020 Apr 2;9(4):158. doi: 10.3390/antibiotics9040158 (PMC7235734; doi:10.3390/antibiotics9040158)
Supplement: Supplementary file 1 [file antibiotics-09-00158-s001.zip › Supplementary File S2 - full AMS implementation questionnaire...docx]

**Supplementary 1. Full AMS implementation questionnaire**

# Page 3- Role-specific information

## ASK ALL:

1. In terms of organisation structure, where does your medicines management team sit? Please select one

-Our medicines management team sits within the CCG and covers the following CCGs

-Our medicines management team sits within the CSU and covers the following CCGs

**🡪If CSU, please specify which CSU** (Comments box)

-Our medicines management team sits within ‘Other’ and covers the following CCGs

**🡪If other, please specify** (Comments box)

## ASK ALL:

4. Which CCG(s) do you represent?* Please select one CCG from each drop-down. Leave the remaining drop-downs blank once you have selected all of the CCGs you represent

🞏Dropdown of all 209 CCGs

🞏 Dropdown of all 209 CCGs

🞏 Dropdown of all 209 CCGs

🞏 Dropdown of all 209 CCGs

🞏 Dropdown of all 209 CCGs

🞏 Dropdown of all 209 CCGs

## ASK ALL:

10. Other CCGs you represent, please specify (Comments box)

# Page 4

## If represent more than one CCG in Q4, ask:

11. You have indicated that you represent more than one CCG. Please choose ONE of the CCGs you represent to complete this questionnaire on behalf of* (Please select one)

🞏Dropdown of all CCGs they have indicated that they represent

## If represent more than one CCG in Q4, ask:

12. AND choose one of the following options: (Please select one)

-I will complete a separate questionnaire for each CCG that I represent

- I will forward the questionnaire on to other contact(s) to complete the questionnaire on behalf of the other CCG(s) that I represent (please provide their contact details for our database)

- I will complete a table at the end of this questionnaire to comment on any differences in AMS activity between each CCG that I represent

## If tick ‘I will forward the questionnaire on to other contact(s) to complete the questionnaire on behalf of the other CCG(s) that I represent (please provide their contact details for our database)’ for Q12, ask:

Q12a. Please provide these contact details (name, email address) for our database:  (Comments boxes)

## ASK ALL:

21. What is your job role? Tick all that apply

-Head of medicines management

-Prescribing advisor

-Pharmacist

-Antimicrobial pharmacist

-Microbiologist

-GP

-Other, please specify (Comments box)

## ASK ALL:

22. May you briefly describe your roles, responsibilities and priorities in relation to antimicrobial stewardship (Comments box)

## ASK ALL:

23. Do you work full-time or part-time? Please tick one

-Full-time

-Part time

🡪 How many hours do you work a week? (Comments box)

## ASK ALL:

24. On average, approximately how many days a month do you spend on antimicrobial stewardship duties? (Assume an average month has a maximum of 22 working days) 
Select whichever best represents you

-Less than 5 days

-5-10 days

-11-15 days

-16-20 days

-21 days or more

## ASK ALL:

25. Comments on your role (Comments box)

# Page 5- TARGET Antibiotics Toolkit


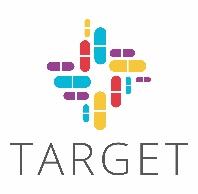


## ASK ALL:

15. Over the last 2 years (2015-2017), have you actively promoted use of the TARGET Antibiotics Toolkit (available on the RCGP website) to support antimicrobial stewardship in primary care? Please tick one

-I’ve never heard of the TARGET Antibiotics Toolkit

-No, we do NOT actively promote the TARGET Antibiotics Toolkit

-Yes, we actively promote the TARGET Antibiotics Toolkit

-Don't know

## ASK ALL:

16. Leaflets to share with patients

Over the last 2 years (2015-2017), which resources from the TARGET Antibiotics Toolkit have you actively promoted for your primary care practitioners to use?


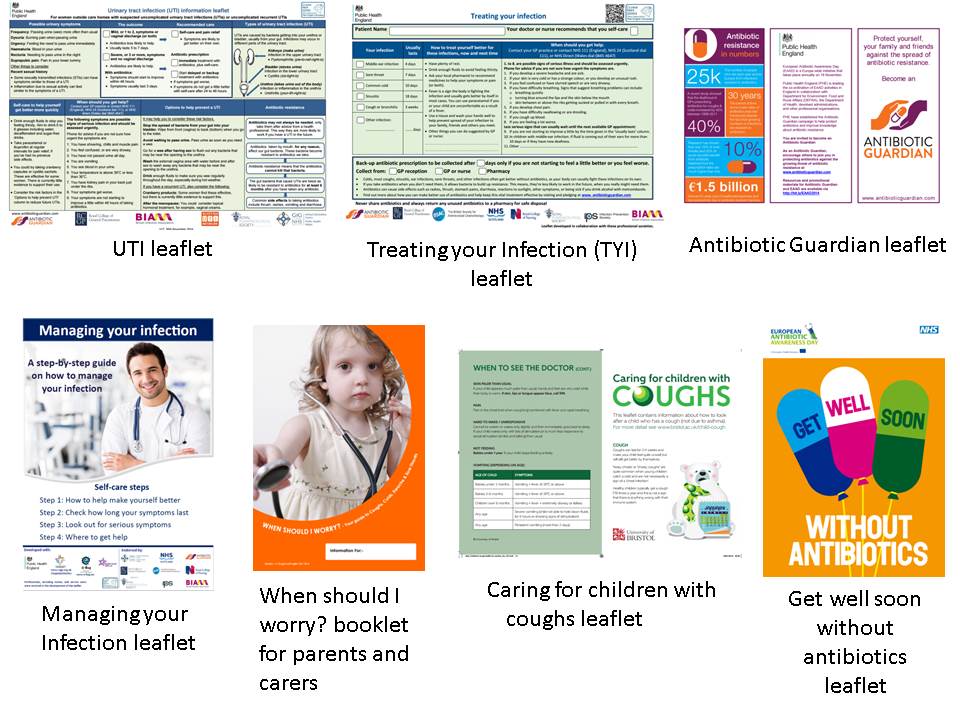


Tick all that apply

-None

-UTI leaflet

-Treating your Infection (TYI) leaflet

-Antibiotic Guardian leaflet

-Managing your Infection leaflet

-When should I worry? booklet for parents and carers

-Caring for children with coughs leaflet

-Get well soon without antibiotics leaflet

-Don’t know

## If tick ‘None’, go to Q.22.

## Unless tick ‘None’ in Q16, ask:

17. What formats have you provided these leaflets in for your primary care practitioners? Tick all that apply for each leaflet


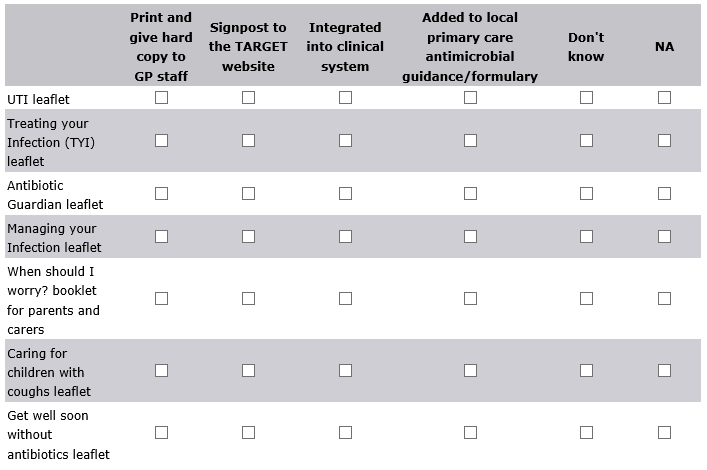


## Unless tick ‘None’ in Q16, ask:

18. Please give details of any other formats that you have provided these leaflets in, specifying format and for which leaflets (Comments box)

## Unless tick ‘None’ in Q16, ask:

19. To which practitioners have you actively promoted these leaflets to? Tick all that apply for each leaflet


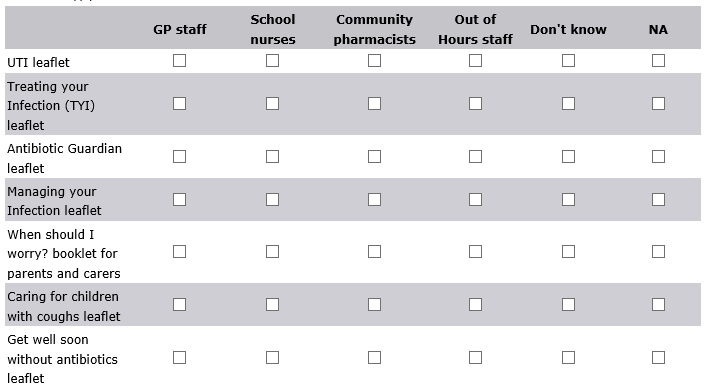


## Unless tick ‘None’ in Q16, ask:

20. Please give details of any other primary care practitioners you have provided these leaflets for, specifying which leaflets and for which primary care practitioners (Comments box)

## Unless tick ‘None’ in Q16, ask:

21. Currently, how is the TARGET Treating Your Infection (TYI) leaflet promoted in your local primary care antimicrobial guidelines?


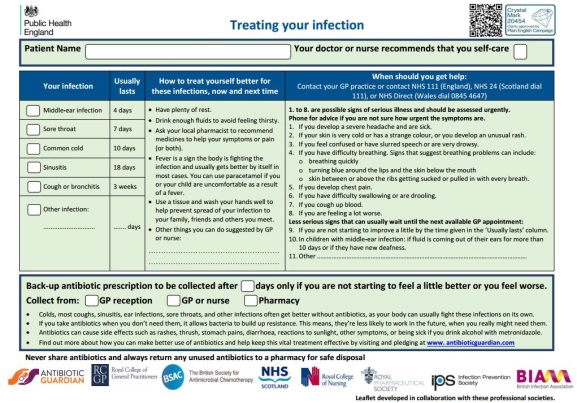


Tick all that apply

-The TYI leaflet is NOT currently promoted in our primary care antimicrobial guidelines

-To support back-up/delayed prescribing

-To support self-care and safety netting

-To support appropriate prescribing

-Don't know

-Other, please specify

## ASK ALL:

22. To what extent do you think the TARGET Antibiotics Toolkit leaflets to share with patients are successful in supporting antimicrobial stewardship in primary care?


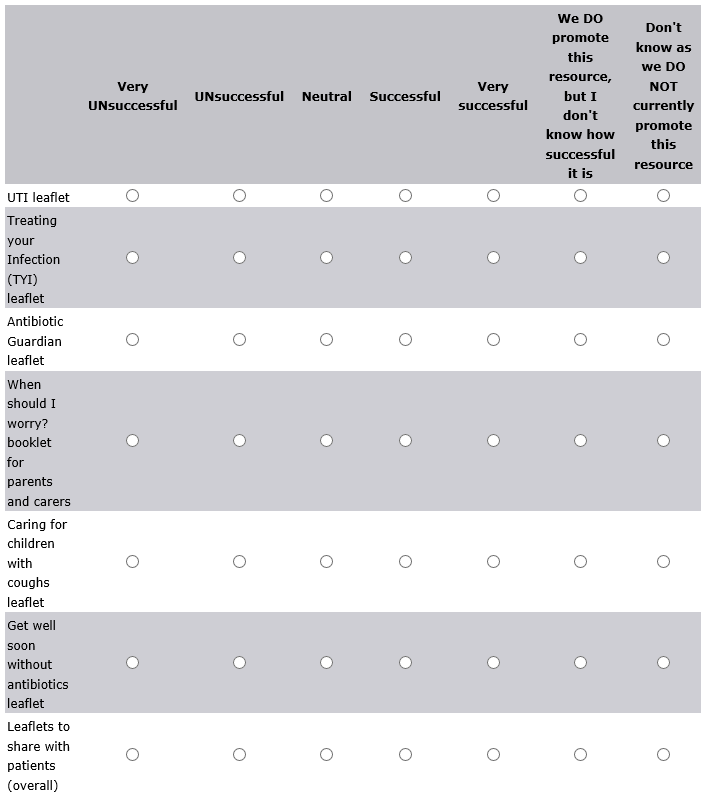


## ASK ALL:

23. Please give a reason for these answers (Comments box)

## ASK ALL:

24. Resources for clinical and waiting areas

Over the last 2 years (2015-2017), which other resources from the TARGET Antibiotics Toolkit have you actively promoted for your primary care practitioners to use?


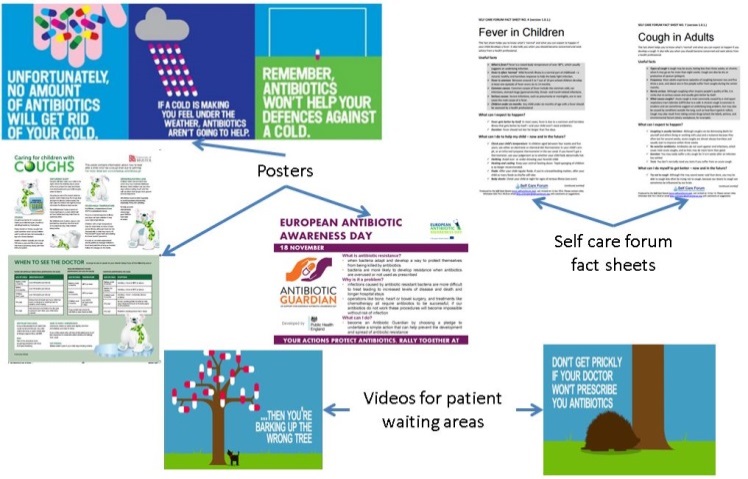


Tick all that apply

-None

-Posters

-Self care forum fact sheets

-Videos for patient waiting areas

-Don’t know

## ASK ALL:

25. To what extent do you think the TARGET Antibiotics Toolkit resources for clinical and waiting areas are successful in supporting antimicrobial stewardship in primary care?

Tick one for each resource


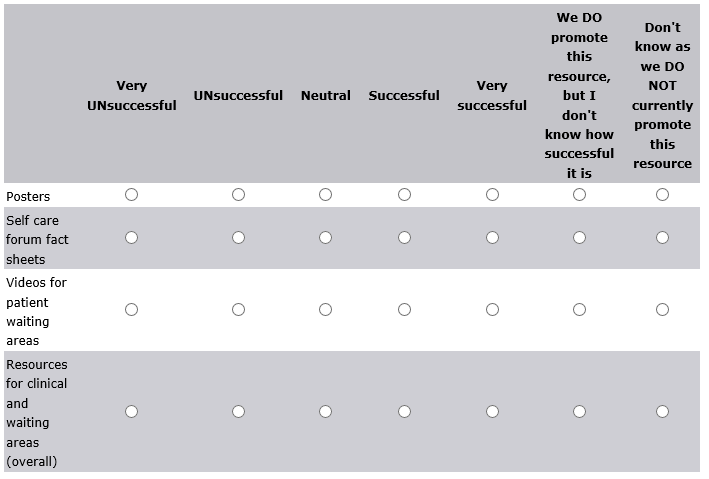


## ASK ALL:

26. Please give a reason for these answers (Comments box)

## ASK ALL:

27. Self assessment checklist for primary care prescribers

Over the last 2 years (2015-2017), have you actively promoted use of the TARGET Antibiotics Toolkit self-assessment checklist for primary care prescribers?


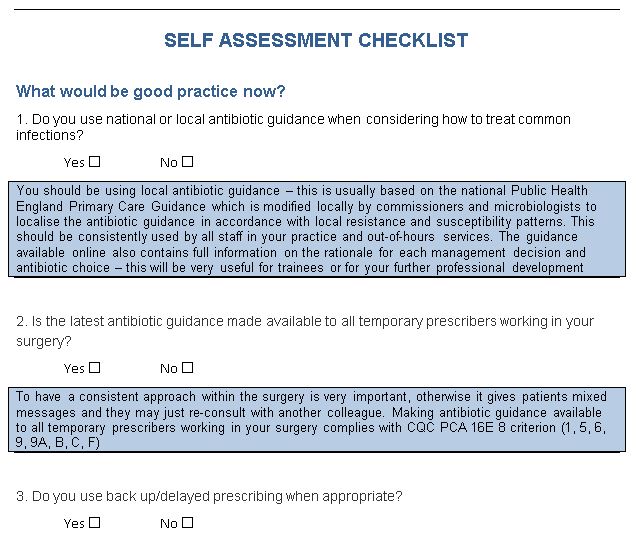


(checklist to measure prescribers’ current antibiotic prescribing practice against others in their region and nationally) Please tick one

-No

-Yes

-Don't know

## ASK ALL:

28. To what extent do you think the TARGET Antibiotics Toolkit self-assessment checklist for primary care prescribers is successful in supporting antimicrobial stewardship in primary care? Please tick one

##
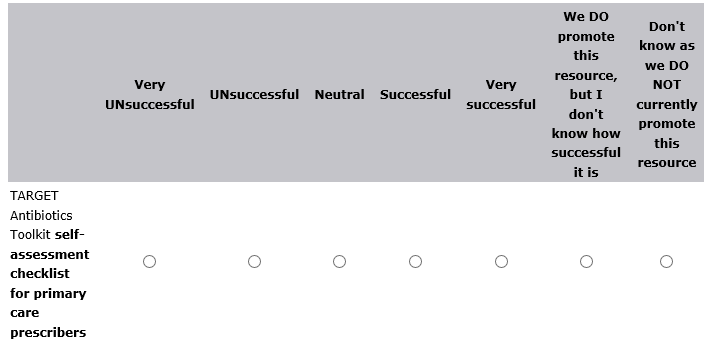


## ASK ALL:

29. Please give a reason for this answer (Comments box)

## ASK ALL:

30. Approximately what percentage of primary care practitioners use the TARGET Antibiotic Toolkit resources?

<5%

25%

50%

75%

>90%

Don't know

## ASK ALL:

30a. How do you monitor this? (Comments box)

## ASK ALL:

31. Any further comments on TARGET Antibiotic resources for practitioners (Comments box)

## ASK ALL:

32. Resources for commissioners

As a commissioner, over the last 2 years (2015-2017), which resources from the TARGET Antibiotics toolkit have you used yourself? Tick all that apply


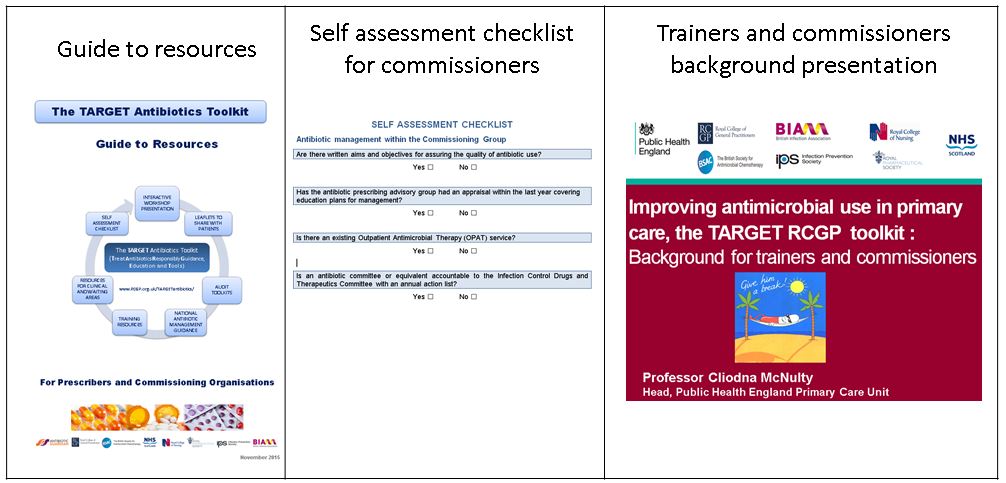


-None

-Guide to resources

-Self assessment checklist for commissioners

-Trainers and commissioners background presentation

## ASK ALL:

33. Over the last 2 years (2015-2017), have you used any other tools to make any assessment of current antimicrobial stewardship initiatives across your CCG(s)?


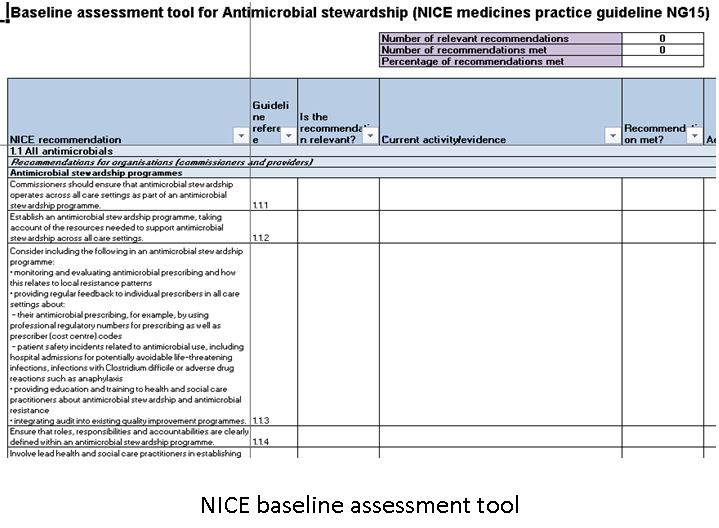


Tick all that apply

-No, we don’t use anything else to assess our AMS initiatives across our CCG(s)

-Yes, we use the NICE baseline assessment tool

-Yes, we use 'other' tools to assess our AMS initiatives across our CCG(s)

- Please specify 'other' tools (Comments box)

-Don't know

## Unless tick ‘None’ in Q32., ask:

34. When was the last time you used these resources for commissioners? Tick one for each resource


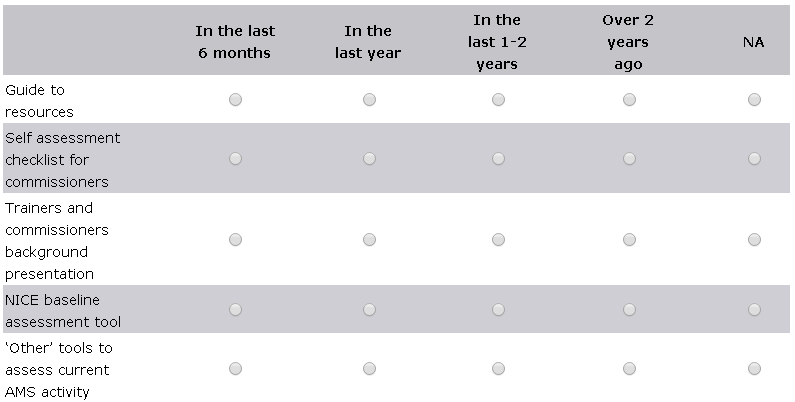


## If tick ‘Self assessment checklist for commissioners’ in Q32., ask:

35. To what extent do you think the TARGET Antibiotics self-assessment checklist for commissioners is successful in supporting antimicrobial stewardship in primary care? Please tick one

##
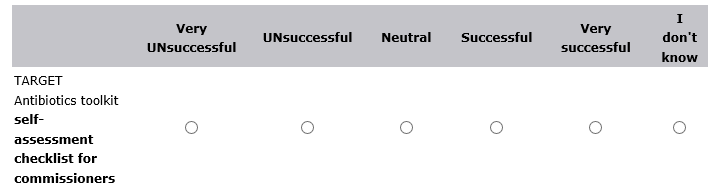


## If tick ‘Self assessment checklist for commissioners’ in Q32., ask:

36. Please give a reason for this answer (Comments box)

## ASK ALL:

37. Any further comments on TARGET Antibiotics resources for commissioners (Comments box)

## ASK ALL:

38. Any further comments on the TARGET Antibiotics Toolkit, overall (Comments box)

# Page 6- Antimicrobial guidance/formulary

**The following page will ask about your primary care antimicrobial guidance and any of PHE’s quick reference diagnostic guides that you promote**

## ASK ALL:

39. Does your CCG/CSU have an antimicrobial committee or equivalent process that develops and reviews local antimicrobial guidelines for primary care? Please tick one

-No

-Yes

-Don't know

## ASK ALL:

40. How frequently are your local primary care antimicrobial guidelines reviewed? Please tick one

-Currently being updated

-Annually

-Every 2 years

-Every 3 years

-Don't know

-Other, please specify (Comments box)

## ASK ALL:

41. What antimicrobial guidelines do you promote for your primary care clinicians to use? Tick all that apply

-Signpost to Public Health England (PHE) managing common infections guidance

-Modified/localised guidance based on PHE managing common infections guidance

-Develop localised guidance from scratch

-Modified local secondary care guidelines

-Don’t know

-Other, please specify (Comments box)

## If tick ‘Modified/localised guidance based on PHE managing common infections guidance’ in Q41., ask:

42. When last updated, what changes did the development group make to the PHE managing common infections guidance in the development of your local primary care antimicrobial prescribing guidance? Tick all that apply

-Added local telephone numbers but change very little else

-Added extra conditions / sections

-What conditions/sections have you added? (Comments box)

-Removed conditions / sections

-What conditions/sections have you removed? (Comments box)

-Changed diagnostic advice

-What diagnostic advice have you changed? (Comments box)

-Reformat layout

-Don’t know

-Other, please specify (Comments box)

## If tick ‘Modified/localised guidance based on PHE managing common infections guidance’ in Q41., ask:

46. How useful is the PHE managing common infections guidance in facilitating the creation of your guidance?

-Not at all useful

-Not useful

-Neutral

-Useful

-Very useful

-Don’t know

## If tick ‘Modified/localised guidance based on PHE managing common infections guidance’ or ‘Signpost to Public Health England (PHE) managing common infections guidance ‘ in Q41., ask:

47. Please state two strengths of the PHE managing common infections guidance (Comments box)

## If tick ‘Modified/localised guidance based on PHE managing common infections guidance’ or ‘Signpost to Public Health England (PHE) managing common infections guidance ‘ in Q41., ask:

48. Please state two weaknesses of the PHE managing common infections guidance (Comments box)

## If tick ‘Modified/localised guidance based on PHE managing common infections guidance’ or ‘Signpost to Public Health England (PHE) managing common infections guidance ‘ in Q41., ask:

49. Are there any significant clinical areas that you think should be included in the PHE managing common infections guidance? (PHE recently added: scarlet fever; genital herpes; gonorrhoea; recurrent vaginal candidiasis; acne; erysipelas; tick bites (Lyme disease); mastitis; blepharitis) (Comments box)

## ASK ALL:

50. Over the last 2 years (2015-2017), in which format has the primary care antimicrobial prescribing guidance been presented to primary care practitioners?

-Electronic format on intranet

-Electronic format on internet

-Included in local area formulary

-Provide paper copy

-Integrated into the clinical system

-App

- Please provide the name of the app or a link to where it can be downloaded from (Comments box)

-Don’t know

-Other, please specify (Comments box)

## ASK ALL:

51. Since it was last updated, approximately what percentage of primary care practitioners have accessed the primary care antimicrobial guidance you promote? Please tick one

<5%

25%

50%

75%

>90%

Don't know

## ASK ALL:

51a. How do you monitor this? (Comments box)

## ASK ALL:

52. Over the last 2 years (2015-2017), which of PHE’s quick reference diagnostic guides have you promoted for your primary care clinicians to use?

-None

-Urinary tract infections

-Abnormal vaginal discharge

-Chlamydia trachomatis

-Fungal skin and nail infections

-Infectious diarrhoea

-Helicobacter pylori

-Venous leg ulcers

-Methicillin-resistant Staphylococcus aureus (MRSA)

-Panton-Valentine-leukocidin Staphylococcus aureus (PVL-SA)

-Don’t know

## ASK ALL:

53. Are there any additional significant clinical areas that you think should have individual quick reference diagnostic guides that could benefit primary care practitioners? (Comments box)

## ASK ALL:

54. Any further comments on antimicrobial guidance/formulary (Comments box)

# Page 7- Education/training

## ASK ALL:

55. Over the last 2 years (2015-2017), locally, how have primary care practitioners received education/training on AMS? Tick all that apply

-They have not received education/training on AMS

-Face-to-face education/training

-Signposted to e-learning

-Don't know

-Other, please specify (Comments box)

## If tick ‘They have not received education/training on AMS’ in Q55., go to Q66. Otherwise ask:

56. Over the last 2 years (2015-2017), who has been the focus of the AMS education/training? Tick all that apply

-All GP practice staff

-GP practice antimicrobial prescribers

-Out of hours staff

-Care home staff

-Community pharmacists

-Don't know

-Other, please specify (Comments box)

## If tick ‘Face-to-face education/training’ in Q55., ask:

57. Over the last 2 years (2015-2017), who has delivered the face-to-face AMS education/training to primary care practitioners? Tick all that apply

-I have

-CCG prescribing advisor

-CCG practice pharmacist

-NHSE GP practice pharmacist

-Local microbiologist

-NHSE GP practice antimicrobial lead/champion

-Don’t know

-Other, please specify (Comments box)

## If tick ‘Face-to-face education/training’ in Q55., ask:

58. Over the last 2 years (2015-2017), what resources have you/they use in this AMS education/training? Tick all that apply

-Locally developed AMS education/training

-Please specify how your locally developed education/training was developed and which sources/presentations it is based on (Comments box)

-TARGET antibiotic group presentation

-Detailed action planning

-Antimicrobial prescribing data

-Audit tools

-Don't know

-Other, please specify (Comments box)

## If tick ‘Face-to-face education/training’ in Q55., ask:

59. Over the last 2 years (2015-17), approximately, what percentage of primary care practices in your CCG(s) have received face-to-face AMS education/training? Please tick one

<25%

25-50%

51-75%

>75%

Don’t know

## If tick ‘Face-to-face education/training’ in Q55., ask:

60. Any further comments on face-to-face education/training (Comments box)

## If tick ‘Signposted to e-learning’ in Q55., ask:

61. Over the last 2 years (2015-2017), which e-learning have you signposted your primary care practitioners to? Tick all that apply

-BSAC’s MOOC (Massive Open Online Course)

-HEE (Health Education England) ‘Reducing Antimicrobial Resistance: An Introduction’

-NICE AMS course

-e-LfH (eLearning for Healthcare) Level 1 AMR

-TARGET eLearning resources eg. TARGET antibiotic webinar series, TARGET Antibiotic Resistance in Primary Care e-module, Skin Infections online course, MARTI Managing Acute Respiratory Tract Infections, Urinary Tract Infections, Managing infectious diarrhoea, Sexual Health in Primary Care

-CPPE (Centre for Pharmacy Postgraduate Education) resources

-Don't know

-Other, please specify (Comments box)

## If tick ‘TARGET eLearning resources eg. TARGET antibiotic webinar series, TARGET Antibiotic Resistance in Primary Care e-module, Skin Infections online course, MARTI Managing Acute Respiratory Tract Infections, Urinary Tract Infections, Managing infectious diarrhoea, Sexual Health in Primary Care’ in Q61., aks:

62. Over the last 2 years (2015-2017), which TARGET Antibiotics toolkit e-Learning resources have you signposted your primary care practitioners to? Tick all that apply

-TARGET antibiotic webinar series

-TARGET Antibiotic Resistance in Primary Care e-module

-Skin Infections online course

-MARTI Managing Acute Respiratory Tract Infections

-Urinary Tract Infections

-Managing infectious diarrhoea

-Sexual Health in Primary Care

-STAR: Stemming the Tide of Antibiotic Resistance e-module

-National Prescribing Centre (NPC) e-learning

-Don't know

## If tick ‘Signposted to e-learning’ in Q55., ask:

63. Over the last 2 years (2015-2017), have you monitored how many primary care practitioners have completed the e-learning you signpost to?

-No

-Yes

-How do you monitor this? (Comments box)

-Don’t know

## If tick ‘Signposted to e-learning’ in Q55., ask:

64. How have you been promoting e-learning on AMS for your primary care practitioners? eg are you promoting specific webinars? (Comments box)

## If tick ‘Signposted to e-learning’ in Q55., ask:

65. Any further comments on e-learning (Comments box)

## ASK ALL:

66. Over the last 2 years (2015-2017), have you, yourself, accessed/received any education/training on AMS in your role?

-No

-Yes

-Please provide details of any AMS education/training that you have accessed/received (Comments box)

-Don’t know

## ASK ALL:

67. To what extent do you think education/training, generally, is successful in supporting antimicrobial stewardship in primary care?

##
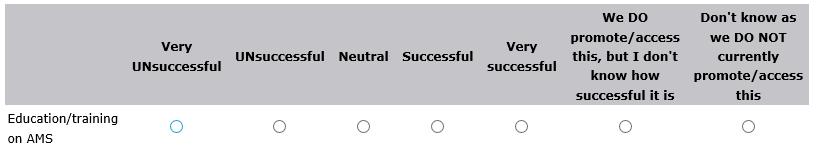


## ASK ALL:

68. Please give a reason for this answer (Comments box)

## ASK ALL:

69. How important are the following characteristics of education/training in relation to facilitators for AMS in your CCG(s)? Tick one for each row

|  |  | **Very UNimportant** |  | **UNimportant** |  | **Neutral** |  | **Important** |  | **Very important** |  |  |
| --- | --- | --- | --- | --- | --- | --- | --- | --- | --- | --- | --- | --- |
| Face-to-face education/training for primary care practitioners |  |  |  |  |  |  |  |  |  |  |  |  |
| Education/training for primary care practitioners delivered by a local/respected expert in the field ie. Antimicrobial pharmacist, microbiologist etc |  |  |  |  |  |  |  |  |  |  |  |  |
| CCG/CSUs to be able to request education/training from a reputable source eg. PHE |  |  |  |  |  |  |  |  |  |  |  |  |
| Mixing up the format of education/training so it seems less repetitive to primary care practitioners |  |  |  |  |  |  |  |  |  |  |  |  |
| Education/training including real-life relatable clinical case studies |  |  |  |  |  |  |  |  |  |  |  |  |
| Education/training that emphasises the link between appropriate antimicrobial prescribing and reducing workload for the practitioner |  |  |  |  |  |  |  |  |  |  |  |  |
| Primary care-specific e-learning |  |  |  |  |  |  |  |  |  |  |  |  |
| Education/training for the whole practice |  |  |  |  |  |  |  |  |  |  |  |  |
| Tailored education/training for antimicrobial prescribers |  |  |  |  |  |  |  |  |  |  |  |  |
| Making AMS education/training mandatory for all primary care practitioners |  |  |  |  |  |  |  |  |  |  |  |  |

## ASK ALL:

70. Please give details of any other facilitators for AMS education/training (Comments box)

## ASK ALL:

71. Any further comments on education/training (Comments box)

# Page 8- Benchmarking and feedback of antimicrobial prescribing data

## ASK ALL:

72. Over the last 2 years (2015-2017), have you fed back local/national antimicrobial prescribing data to the CCG/CSU board? Please tick one

-No

-Why have you not fed back local/national antimicrobial prescribing data to the CCG/CSU board? (Comments box)

-Yes

-Don’t know

## ASK ALL:

73. Over the last 2 years (2015-2017), have you fed back local/national antimicrobial prescribing data to your primary care practitioners? Tick all that apply

-No

-Why have you not fed back local/national antimicrobial prescribing data to your primary care practitioners? (Comments box)

-Yes, to general practices

-Yes, to out of hours services

-Don't know

-Other, please specify (Comments box)

## ASK ALL:

74. Where do you source antimicrobial prescribing data from? Tick all that apply

-NHS BSA ISP (Information Service Portal)

- NHS BSA ePACT

- NHS BSA ePACT2 AMS dashboard

- PHE Portal (Fingertips)

- PrescQIPP AMS Hub

- OpenPrescribing

- Commissioning Support Unit (CSU) reporting

-Don't know

-Other, please specify (Comments box)

## If tick ‘No’ to Q73, skip to Q81. Otherwise ask:

75. What is the nature of the antimicrobial prescribing data that you feed back to primary care practitioners? Tick all that apply

**Antimicrobial data**

-Overall antimicrobial prescribing

-Data on each antimicrobial group

-Data aligned to national AMS initiatives, such as CCG QP (Quality Premium) and IAF (Improvement Assessment Framework) (antimicrobial items/STAR-PU etc)

-Don't know

-Other, please specify (Comments box)

**Level of antimicrobial prescribing data**

- CCG level data

- GP practice level data

- Individual prescribers level data

- STP (Sustainability and Transformation Plan) level data

- ‘Similar 10’ level data

-Don't know

-Other, please specify (Comments box)

**Benchmarking of antimicrobial prescribing data**

-Benchmarking against national average

-Benchmarking against regional average

-Benchmarking against NHSE area average

-Benchmarking against ‘similar 10’ CCGs

-Benchmarking against STP

-Benchmarking against Academic Health Science Networks (AHSN)

-Don't know

-Other, please specify (Comments box)

## Unless tick ‘No’’ to Q73., ask:

78. How often do you feed this antimicrobial prescribing data back to the practitioners and/or CCG/CSU board? Please tick one


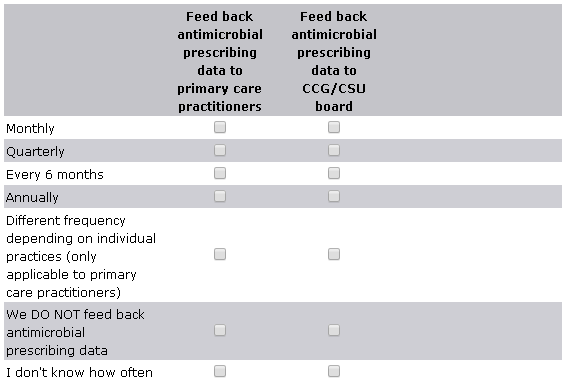


## If tick ‘Different frequency depending on individual practices (only applicable to primary care practitioners) in Q78, ask:

78a. Please specify how frequently you feed this antimicrobial prescribing data back to the practitioners depending on the individual practices (Comments box)

## Unless tick ‘No’’ to Q73., ask:

79. How have you communicated the antimicrobial prescribing data?

Tick all that apply


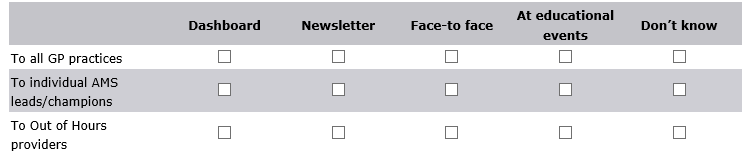


## Unless tick ‘No’’ to Q73., ask:

80. Please give details of any other ways that you have communicated antimicrobial prescribing data with primary care practitioners (Comments box)

## ASK ALL:

81. To what extent do you think feeding back antimicrobial prescribing data is successful at raising your primary care practitioners’ awareness and prioritising AMS activity? Tick one for each row


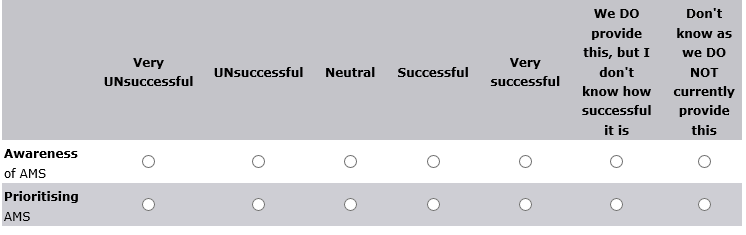


## ASK ALL:

82. Please give a reason for these answers (Comments box)

## ASK ALL:

83. To what extent do you think feeding back antimicrobial prescribing data is successful at raising your CCG/CSU’s awareness and prioritising AMS activity? Tick one for each row


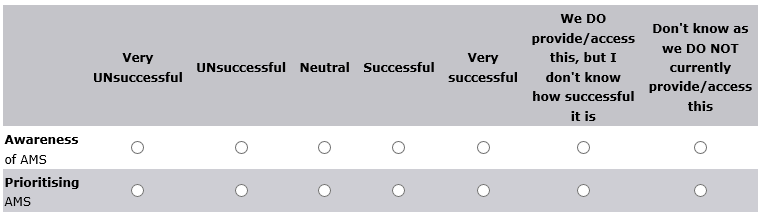


## ASK ALL:

84. Please give a reason for these answers (Comments box)

## ASK ALL:

85. Any further comments on benchmarking and feedback of prescribing data (Comments box)

# Page 9- Audits

Audits aim to provide a snapshot of prescribing at a particular point in time. Conducting audits and action planning together enables a practice to understand current antibiotic prescribing patterns, compliance with guidelines and can be used to discuss within the team and make improvements if necessary.

## ASK ALL:

86. Over the last 2 years (2015-2017), how have audits for AMS been undertaken in your CCG(s)?

-AMS audits have NOT been undertaken in our CCG(s)

-Practice staff have undertaken AMS audits

-CCG staff have undertaken AMS audits on behalf of the practices as part of a CCG workforce programme

-Practice staff have undertaken AMS audits for re-validation purposes

-Trainee GPs have undertaken AMS audits for their practice

-All practices in our CCG(s) have undertaken/been asked to undertake AMS audits

-Specific practices in our CCG(s) have undertaken/been asked to undertake AMS audits

-Don’t know

-Other, please specify (Comments box)

## If tick ‘AMS audits have NOT been undertaken in our CCG(s)’ in Q86, skip to Q95. Otherwise ask:

87. Please give details of how it was decided which practices in your CCG(s) should undertake/were asked to undertake audits (Comments box)

## Unless tick ‘AMS audits have NOT been undertaken in our CCG(s)’ in Q86, ask:

88. Over the last 2 years (2015-2017), which template(s) have you used/promoted for your practices to use to audit antimicrobial use?

-We signpost to the TARGET antibiotic audit templates

-We modify the TARGET antibiotic audit templates and provide this to the practices

-We produce audit tools from scratch

-Don't know

-Other, please specify (Comments box)

## Unless tick ‘AMS audits have NOT been undertaken in our CCG(s)’ in Q86, ask:

89. Complete the following sentence (tick all that apply)

All practices that have undertaken/been asked to undertake an audit...

...take part in the same audit(s)

...choose their own antimicrobial audit topic(s)

Don't know

Other, please specify (Comments box)

## Unless tick ‘AMS audits have NOT been undertaken in our CCG(s)’ in Q86, ask:

90. Over the last 2 years (2015-2017), which conditions have you promoted/undertaken audits in?

| **Upper Respiratory Infections** |
| --- |
| Sore throat |
| Acute Otitis Media |
| **Lower Respiratory Tract Infections** |
| Acute cough/bronchitis |
| Acute exacerbation of COPD |
| Community acquired pneumonia- treatment in the community |
| **Urinary Tract Infections** |
| UTI in adults (lower)- uncomplicated |
| UTI in adults (lower)- >65 years |
| Recurrent UTI in non-pregnant women |
| Acute pyelonephritis |
| **Skin Infections**  Don’t know |

**Other**, please specify (Comments box)

## Unless tick ‘AMS audits have NOT been undertaken in our CCG(s)’ in Q86, ask:

91. Over the last 2 years (2015-2017), what has been audited? Tick all that apply

-Choice of antimicrobial

-Duration of antimicrobial

-Use of delayed/ back-up antimicrobial

-Use of the TRAGET Treating Your Infection (TYI) leaflet

-Diagnosis of infection

-Don't know

-Other, please specify (Comments box)

## Unless tick ‘AMS audits have NOT been undertaken in our CCG(s)’ in Q86, ask:

92. Over the last 2 years (2015-2017), how have audit findings been reported/shared? Tick all that apply

-Audit findings are NOT reported/shared

-Audit findings are discussed in the practice with the prescribers

-Audit findings are discussed in the practice with as many primary care practitioners as possible

-Audit findings are reported to the CCG/CSU

-Audit findings are reported to the CCG/CSU board

-Don't know

-Other, please specify (Comments box)

## Unless tick ‘AMS audits have NOT been undertaken in our CCG(s)’ in Q86, ask:

93. In the last year (2016/17), how frequently did you promote for audits to be undertaken? Please tick one

-Annually

-More than one per year

-Don't know

-Other, please specify (Comments box)

## Unless tick ‘AMS audits have NOT been undertaken in our CCG(s)’ in Q86, ask:

94. In the last year (2016/17), approximately, what percentage of practices have completed at least one AMS audit in your CCG(s)?

0%

1-25%

26-50%

51-75%

76-99%

100%

Don’t know

## If provide an answer to Q94, ask:

94a. How do you know this? (Comments box)

## ASK ALL:

95. To what extent do you think audits are successful in supporting antimicrobial stewardship in primary care? Please tick one

##
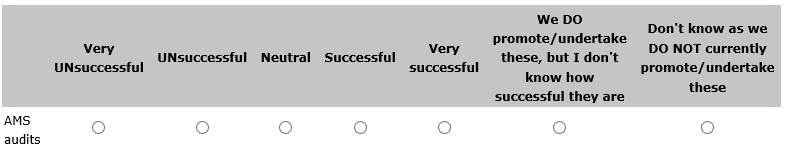


## ASK ALL:

96. Please give a reason for this answer (Comments box)

## ASK ALL:

97. Any further comments about audits (Comments box)

Well done, you've reached the half way point marker! Good news is that the questions get shorter from here onwards!

# Page 10- NHS Quality Premium for AMR

The Quality Premium is intended to reward clinical commissioning groups (CCGs) for improvements in the quality of the services that they commission and for associated improvements in health outcomes.

## ASK ALL:

98. 2 years ago (2015/16), which QP targets did your CCG(s) reach? Tick all that apply

-I've never heard of the Quality Premium

## -We did not attempt to reach either of the targets for the 2015/16 QP

## - We attempted, but did not achieve either of the targets for the 2015/16 QP

## - Reduction in the number of antibiotics prescribed in primary care by 1% (or greater) from each CCG’s 2013/14 value. Individual practice reduction to be agreed by the CCG with each practice

## - Reduction in the proportion of broad spectrum antibiotics prescribed in primary care (number of co-amoxiclav, cephalosporins and quinolones as a percentage of the total number of selected antibiotics prescribed in primary care to be reduced by 10% from each CCG’s 2013/14 value, or to be below the 2013/14 median proportion for English CCGs (11.3%), whichever represents the smallest reduction for the CCG in question)

## - Don't know

## If tick ‘I've never heard of the Quality Premium’ in Q98, skip to Q111.

## If tick anything other than ‘I've never heard of the Quality Premium’ or ‘We did not attempt to reach either of the targets for the 2015/16 QP’ in Q98, ask:

99. Briefly describe what you did to try and reach these 2015/16 targets (Comments box)

## If tick ‘We did not attempt to reach either of the targets for the 2015/16 QP’ in Q98, ask:

100. Why did you not attempt to reach either of the QP targets in 2015/16? (Comments box)

## Unless tick ‘I've never heard of the Quality Premium’ in Q98, ask:

101. Last year (2016/17), which QP targets did your CCG(s) reach? Tick all that apply

- We did not attempt to reach either of the targets for the 2016/17 QP

- We attempted, but did not achieve either of the targets for the 2016/17 QP

-Reduction in the number of antibiotics prescribed in primary care. The required performance in 2016/17 must either be: a 4% (or greater) reduction on 2013/14 performance; OR equal to (or below) the England 2013/14 mean performance of 1.161 items per STAR-PU

-Reduction in the proportion of broad spectrum antibiotics prescribed in primary care (number of co-amoxiclav, cephalosporins and quinolones as a proportion of the total number of selected antibiotics prescribed in primary care to either: to be equal to or lower than 10%; or to reduce by 20% from each CCG’s 2014/15 value

-Don’t know

## If tick anything other than ‘We did not attempt to reach either of the targets for the 2016/17 QP’ in Q101, ask:

102. Briefly describe what you did to try and reach these 2016/17 targets (Comments box)

## If tick ‘We did not attempt to reach either of the targets for the 2016/17 QP’ in Q101, ask:

103. Why did you not attempt to reach either of the QP targets in 2016/17? (Comments box)

## Unless tick ‘I've never heard of the Quality Premium’ in Q98, ask:

104. This year (2017/18), which QP targets is your CCG/CSU attempting to reach? Tick all that apply

-We are not attempting to reach the targets

-Part a) reducing gram negative blood stream infections (BSI) across the whole health economy (a 10% reduction (or greater) in all E coli BSI reported at CCG level based on 2016 performance data AND collection and reporting of a core primary care data set for all E coli BSI in Q2-4 2017/18)

-Part b) reduction of inappropriate antibiotic prescribing for urinary tract infections (UTI) in primary care (a 10% reduction (or greater) in the Trimethoprim: Nitrofurantoin prescribing ratio based on CCG baseline data (June15-May16) AND a 10% reduction (or greater) in the number of trimethoprim items prescribed to patients aged 70 years or greater on baseline data (June15-May16))

-Part c) sustained reduction of inappropriate prescribing in primary care (items per STAR-PU must be equal to or below England 2013/14 mean performance value of 1.161 items per STAR-PU)

-Don't know

## If tick anything other than ‘We are not attempting to reach the targets’ in Q104, ask:

105. Briefly describe what you are doing to try and reach these 2017/18 targets (Comments box)

## If tick ‘We are not attempting to reach the targets’ in Q104, ask:

106. Why are you not attempting to reach any of the QP targets for 2017/18? (Comments box)

## Unless tick ‘I've never heard of the Quality Premium’ in Q98, ask:

107. How successful were the 2015/16 and 2016/17 Quality Premiums at raising your primary care practitioners’ awareness and prioritising AMS activity? Tick one for each row


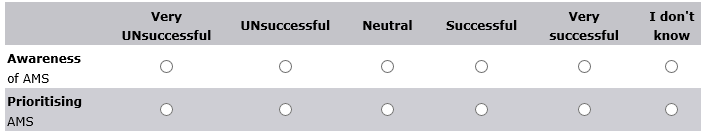


## Unless tick ‘I've never heard of the Quality Premium’ in Q98, ask:

108. Please give a reason for these answers (Comments box)

## Unless tick ‘I've never heard of the Quality Premium’ in Q98, ask:

109. How successful is the Quality Premium at raising your CCG/CSU's awareness and prioritising AMS activity? Tick one for each row


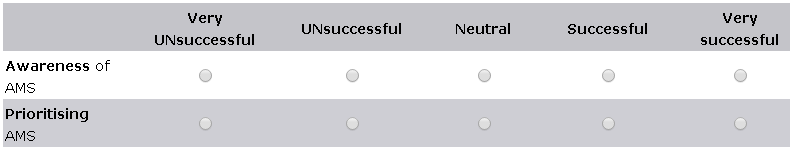


## Unless tick ‘I've never heard of the Quality Premium’ in Q98, ask:

110. Please give a reason for these answers (Comments box)

## ASK ALL:

111. Any further comments on the Quality Premium (Comments box)

# Page 11 – Incentive/Reward schemes

## ASK ALL:

112. Over the last 2 years (2015-2017), what have been/are the components of your incentive/reward scheme for primary care practitioners intended to improve antimicrobial stewardship? Tick all that apply

-We do NOT have an incentive/reward scheme

-Practitioners must complete a self-assessment checklist

-Practices must undertake an audit

-Practitioners must attend/access some form of education/learning on AMR/AMS

-Practitioners are encouraged to become antibiotic guardians

-Don’t know

-Other, please specify (Comments box)

## ASK ALL:

113. Can you briefly describe how your incentive/reward scheme works and/or any other mechanisms you use to engage primary care practitioners in AMS? Including what the incentive/reward is. (Comments box)

## If tick ‘We do NOT have an incentive/reward scheme’ in Q112, skip to Q117. Otherwise ask:

114. How successful is your incentive/reward scheme at raising awareness and prioritising AMS activity for your primary care practitioners? Tick one for each row


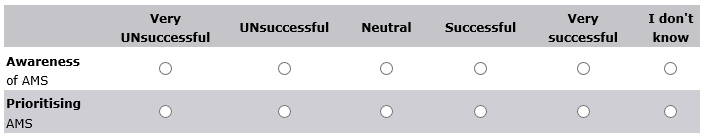


## Unless tick ‘We do NOT have an incentive/reward scheme’ in Q112, ask:

115. Please give a reason for these answers (Comments box)

## Unless tick ‘We do NOT have an incentive/reward scheme’ in Q112, ask:

116. Approximately, what percentage of your primary care practices reached the targets for 2015/16 for your incentive/reward scheme?

0%

1-25%

26-50%

51-75%

76-99%

100%

Don’t know

## ASK ALL:

117. Any further comments on incentive/reward schemes (Comments box)

# Page 12- Antibiotic Guardian Campaign

Antibiotic Guardian, a campaign led by Public Health England (PHE), urges members of the public and healthcare professionals to take action in helping to slow antibiotic resistance and ensure our antibiotics work now and in the future.

To become an Antibiotic Guardian, people choose one pledge about how they can personally prevent infections and make better use of antibiotics and help protect these vital medicines.


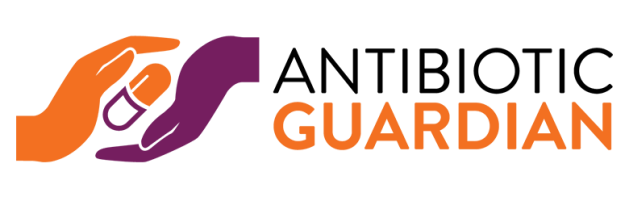


## ASK ALL:

118. Over the last 2 years (2015-2017), how have you actively promote the antibiotic guardian campaign?

-I’ve never heard of the Antibiotic Guardian Campaign

-We do NOT actively encourage people to become antibiotic guardians

-Encourage primary care practitioners to become antibiotic guardians

- Please specify how you encourage primary care practitioners to become antibiotic guardians (Comments box)

-Encourage patients and the public to become antibiotic guardians

- Please specify how you encourage patients and the public to become antibiotic guardians (Comments box)

-Don’t know

-Other, please specify (Comments box)

## If tick ‘I’ve never heard of the Antibiotic Guardian Campaign’, skip to Q123. Otherwise ask:

121. To what extent do you think the antibiotic guardian campaign is successful at raising awareness and prioritising AMS activity for your primary care practitioners? Tick one for each row


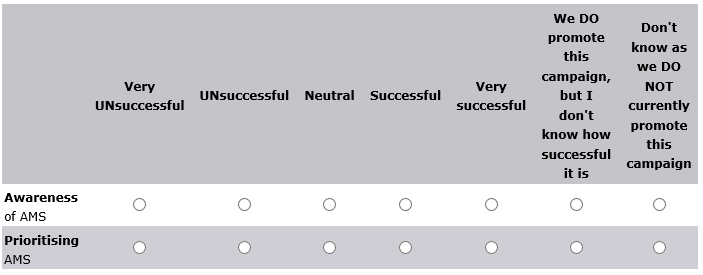


## Unless tick ‘I’ve never heard of the Antibiotic Guardian Campaign’ in Q123, ask:

122. Please give a reason for these answers (Comments box)

## ASK ALL:

123. Any further comments on the antibiotic guardian campaign (Comments box)

# Page 13- Sustainability and Transformation Plans

The NHS and local councils have come together in 44 areas covering all of England to develop proposals and make improvements to health and care. These proposals, called sustainability and transformation plans (STPs), are place-based and built around the needs of the local population.

## ASK ALL:

124. Which STP footprint (geographical area) is your organisation involved with? Please select one

Dropdown of all STPs

Don’t know

## ASK ALL:

125. Were you involved in the development of this STP? Please tick one

-Yes

-No

## ASK ALL:

126. Is AMR/AMS a component of your STP? Please tick one

-Yes

-No

-Don’t know

## If tick ‘No’ in Q126, skip to Q131. Otherwise, ask:

127. What AMR/AMS related content is in your STP? (Comments box)

## Unless tick ‘No’ in Q126, ask:

128. Please describe planned activity (Comments box)

## Unless tick ‘No’ in Q126, ask:

129. What mechanism exists for reporting AMR/AMS at STP level? (Comments box)

## Unless tick ‘No’ in Q126, ask:

130. Do you have a specific person who leads on AMR/AMS at STP level? Please tick one

-Yes

-Who? Please specify job role if known

-No

-Don’t know

## ASK ALL:

131. Any further comments on your local STP (Comments box)

# Page 14- Other resources not previously mentioned

## ASK ALL:

132. Please list up to three other AMS resources/interventions/tools used in primary care

Option 1. (Comments box)

Option 2. (Comments box)

Option 3. (Comments box)

## If no answer is provided to any options in Q132, skip to Q147.

## If provide an answer to Q132 Option 1. Ask:

135. To what extent do you think ##pipeoption1## is successful at raising awareness and prioritising AMS activity for your primary care practitioners? Tick one for each row


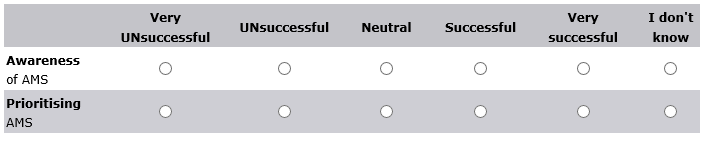


## If provide an answer to Q132 Option 1. Ask:

136. Please give a reason for these answers (Comments box)

## If provide an answer to Q132 Option 1. Ask:

137. Do you monitor how many primary care practitioners use ##pipeoption1##?

-Yes

-How do you monitor this? (Comments box)

-No

-Don’t know

## If provide an answer to Q132 Option 1. Ask:

138. Any further comments on ##pipeoption1## (Comments box)

## If provide an answer to Q132 Option 2. Ask:

139. To what extent do you think ##pipeoption2## is successful at raising awareness and prioritising AMS activity for your primary care practitioners? Tick one for each row


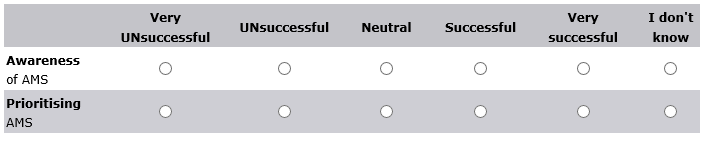


## If provide an answer to Q132 Option 2. Ask:

140. Please give a reason for these answers (Comments box)

## If provide an answer to Q132 Option 2. Ask:

141. Do you monitor how many primary care practitioners use ##pipeoption2##?

-Yes

-How do you monitor this? (Comments box)

-No

-Don’t know

## If provide an answer to Q132 Option 2. Ask:

142. Any further comments on ##pipeoption2## (Comments box)

## If provide an answer to Q132 Option 3. Ask:

143. To what extent do you think ##pipeoption3## is successful at raising awareness and prioritising AMS activity for your primary care practitioners? Tick one for each row


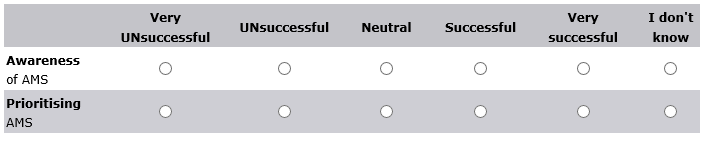


## If provide an answer to Q132 Option 3. Ask:

144. Please give a reason for these answers (Comments box)

## If provide an answer to Q132 Option 3. Ask:

145. Do you monitor how many primary care practitioners use ##pipeoption3##?

-Yes

-How do you monitor this? (Comments box)

-No

-Don’t know

## If provide an answer to Q132 Option 3. Ask:

146. Any further comments on ##pipeoption3## (Comments box)

## ASK ALL:

147. Any further comments on additional resources for AMS (Comments box)

# Page 15- Response to Chief Medical Officer (CMO)’s letter

In Jan 2016 and April 2017, the Chief Medical Officer (CMO) wrote to all GPs working in GP practices that had been identified as prescribing high volumes of antibiotics; practices benchmarked in the top 20% of all GP practices in England for antibacterial Items/STAR-PU. The letter stated that the practice was prescribing antibiotics at a higher rate than 80% of practices in England and provided a leaflet on antibiotics for use with patients.

## ASK ALL:

148. Did any of the practices in your CCG(s) receive a letter from the Chief Medical Officer (CMO) in Jan 2016 and/or April 2017? Please tick one for each row

##
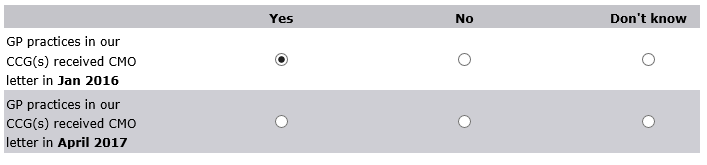


## If tick ‘Yes’ to either option in Q148, ask:

150. What impact did the CMO letter(s) have on the GP practices? Tick all that apply

-Don’t know as have not discussed with the practice staff

-GP practice(s) contacted CCG/CSU for AMS support

-GP practices expressed that they were enthusiastic about receiving a CMO letter

-GP practice(s) expressed concern about receiving a CMO letter

-GP practice(s) expressed anger about receiving a CMO letter

-GP practice(s) stated that they would disregard CMO letter

-Other, please specify (Comments box)

## If tick ‘Yes’ to either option in Q148, ask:

151. How successful was the CMO’s letter(s) at raising awareness and prioritising AMS activity for your primary care practitioners? Tick one for each row


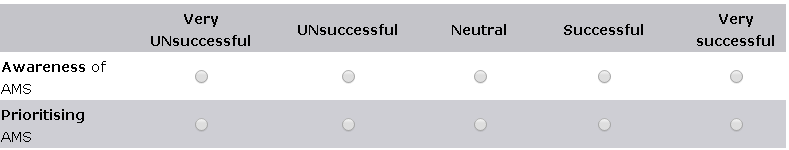


## If tick ‘Yes’ to either option in Q148, ask:

152. Please give a reason for these answers (Comments box)

# Page 16

## ASK ALL:

153. Over the last 2 years (2015-2017), have you promoted back-up/ delayed antibiotic prescribing locally?

-No, we do NOT promote back-up/delayed antibiotic prescribing locally

-Yes, but we do not specify for which conditions

-Yes, for ALL uncomplicated upper respiratory tract infections

-Yes, for acute sore throat

-Yes, for acute otitis media

-Yes, for acute rhinosinusitis

-Yes, for acute cough & bronchitis

-Yes, for uncomplicated lower UTI in adults

-Don’t know

-Other, please specify (Comments box)

## If tick any options that begin with ‘yes’ in Q153, ask:

153a. How do you promote back-up/delayed antibiotic prescribing for these conditions? (Comments box)

## ASK ALL:

154. Do your primary care practitioners participate in any specific AMS campaigns and which resources do you/they use for these? Tick all that apply


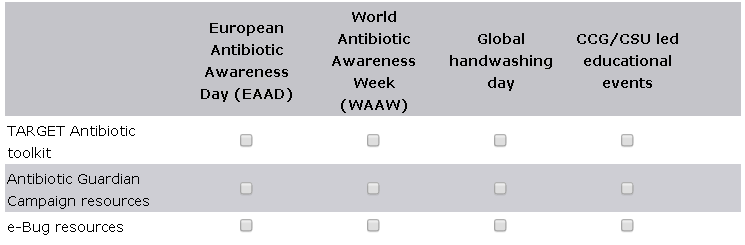


## ASK ALL:

155. How important are national campaigns to educate the general public/patients in relation to facilitators for AMS in your CCG(s)?? Please tick one

##
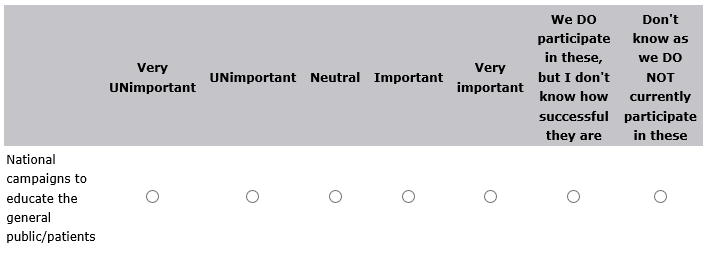


## ASK ALL:

156. Overall, to what extent do you think your primary care practitioners are aware/prioritise/implement AMS activity promoted in your CCG(s)? Tick one for each row


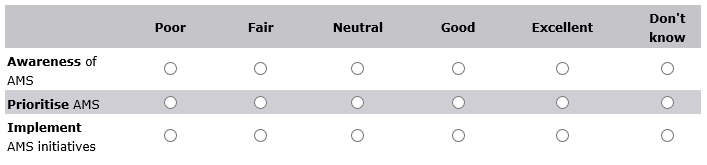


## ASK ALL:

157. Please give a reason for these answers (Comments box)

## ASK ALL:

158. How big a barrier of AMS are the following characteristics for your primary care practitioners? Tick one for each row


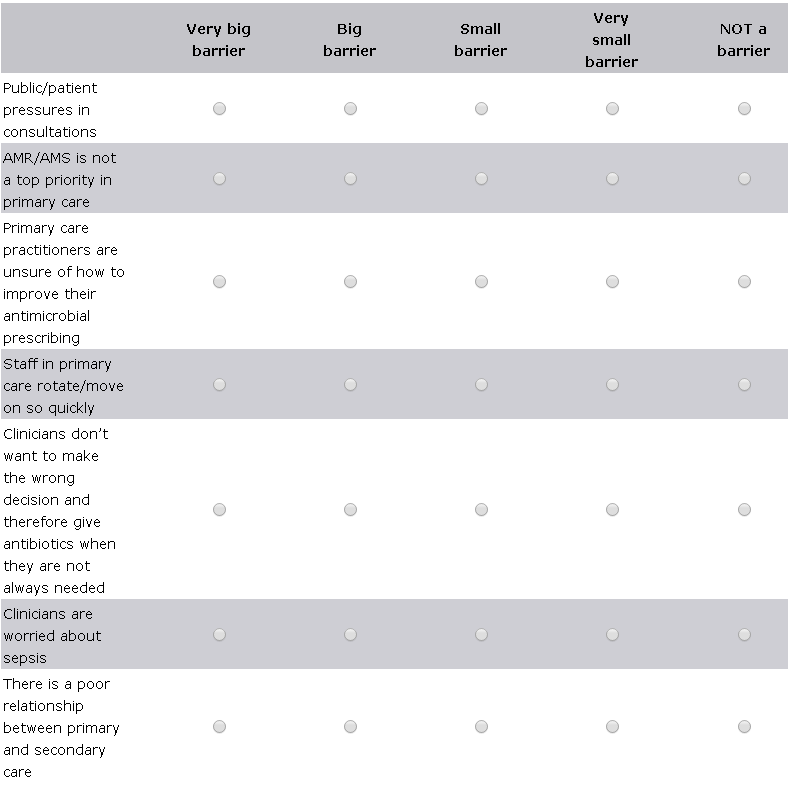


## ASK ALL:

159. Please give details of any other barriers of AMS for your primary care practitioners (Comments box)

## ASK ALL:

160. How important are the following characteristics in relation to facilitators for AMS in your CCG(s)?? Tick one for each row


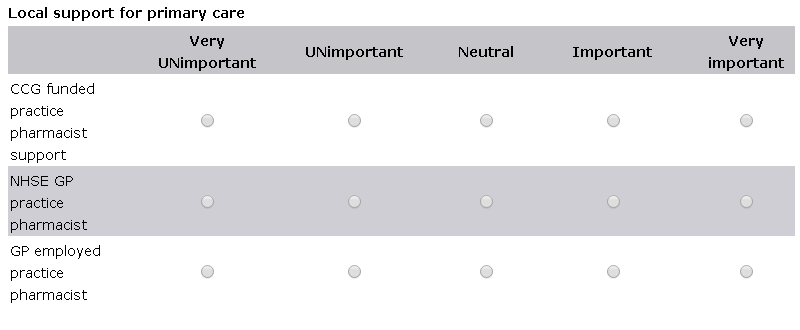


## ASK ALL:

161. Please give details of any other facilitators of AMS for your primary care practitioners (Comments box)

## ASK ALL:

162. Overall, to what extent do you think your medicines management team is aware/prioritises/promotes AMS activity? Tick one for each row


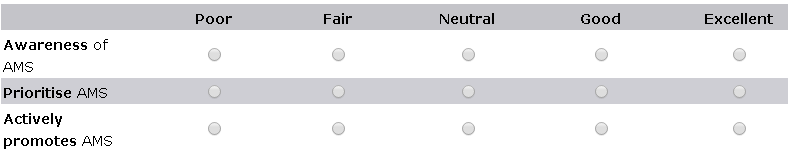


## ASK ALL:

163. Please give a reason for these answers (Comments box)

## ASK ALL:

164. How big a barrier of AMS are the following characteristics for your medicines management team? Tick one for each row


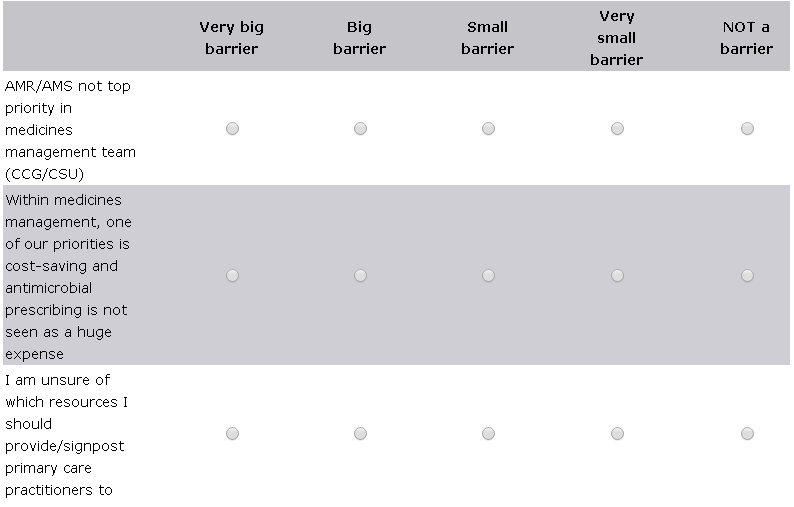


## ASK ALL:

165. Please give details of any other barriers of AMS for your medicines management team (Comments box)

## ASK ALL:

166. How important are the following characteristics in relation to facilitators for AMS in your CCG(s)?? Tick one for each row


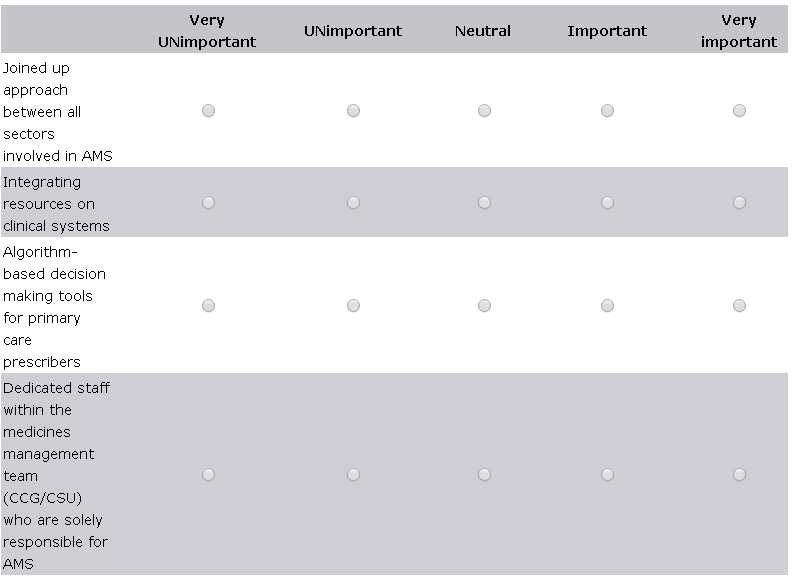


## ASK ALL:

167. Please give details of any other facilitators of AMS for your medicines management team (Comments box)

## ASK ALL:

168. Final comments (Comments box)

# Page 19- Background Information

## ASK ALL:

169. What is your gender? Please tick one

-Male

-Female

-I would rather not say

## ASK ALL:

170. What is your age? (years) Please tick one

-24 or below

-25-35

-36-45

-46-55

-56 or over

-I would rather not say

## ASK ALL:

171. How long have you worked in AMS?

-Less than 1 year

-1-4 years

-5-9 years

-Over 10 years
