# Supplementary material for: What Resources Do NHS Commissioning Organisations Use to Support Antimicrobial Stewardship in Primary Care in England?
Source: Antibiotics (Basel). 2020 Apr 2;9(4):158. doi: 10.3390/antibiotics9040158 (PMC7235734; doi:10.3390/antibiotics9040158)
Supplement: Supplementary file 1 [file antibiotics-09-00158-s001.zip › Supplementary File S1 - full breakdown of responses to each question.docx]

## Supplementary S1. Breakdown of participants responses to each question, including ‘blanks’ and ‘don’t know’.

## In terms of organisation structure, where does your medicines management team sit? n=187

| **Option** | **Number of responses** | **Percentage (%)(n=187)** |
| --- | --- | --- |
| within the CCG | 153 | 82% |
| within the CSU | 23 | 12% |
| Other | 11 | 6% |
| **Total that completed question** | **187** |  |

## Breakdown of the number of responses to each section of total questionnaire

| **Section of questionnaire** | **Number of responses (% of total n=187)** |
| --- | --- |
| Role-specific information | 187 (100%) |
| TARGET Antibiotics Toolkit | 186 (99%) |
| Antimicrobial guidance/formulary | 181 (97%) |
| Education/training | 175 (94%) |
| Benchmarking and feedback of antimicrobial prescribing data | 169 (90%) |
| Audits | 167 (89%) |
| NHS Quality Premium for AMR |  |
| Incentive/reward schemes | 167 (89%) |
| Antibiotic Guardian Campaign | 167 (89%) |
| Sustainability and Transformation Plans (STPs) | 167 (89%) |
| Other resources | 70 (37%) |
| Response to Chief Medical Officer (CMO)'s letter | 166 (89%) |
| Barriers and facilitators | 166 (89%) |

# AMS Education/training

## Over the last 2 years (2015-2017), locally, how have primary care practitioners received education/training on AMS? (n=187)

| **Option** | **Number of CCGs (n)** | **Percentage (%) (n=168)** |
| --- | --- | --- |
| Face-to-face education/training | 140 | 83% |
| Signposted to e-learning | 121 | 72% |
| Other | 22 | 13% |
| **Total that have received education/training** | **168** |  |
| They have not received education/training on AMS | 1 |  |
| Don't know | 6 |  |
| **Total that completed question** | **175** |  |
| Blank | 12 |  |

## Over the last 2 years (2015-2017), who has been the focus of the AMS education/training? (n=174. Not including the one CCG that reported that ‘They have not received education/training on AMS’ or the 12 CCGs that left blank)

| **Option** | **Number of CCGs (n)** | **Percentage (%) (n=166)** |
| --- | --- | --- |
| All GP practice staff | 69 | 42% |
| GP practice antimicrobial prescribers | 112 | 67% |
| Out of hours staff | 46 | 28% |
| Care home staff | 41 | 25% |
| Community pharmacists | 30 | 18% |
| Other | 21 | 13% |
| **Total that reported AMS education/training focus** | **166** |  |
| Don't know | 3 |  |
| **Total that completed question** | **169** |  |
| Blank | 5 |  |

## Over the last 2 years (2015-2017), who has delivered the face-to-face AMS education/training to primary care practitioners? (n=140. Those who indicated that their primary care practitioners had received face-to-face AMS education/training in the last two years (2015-2017))

| **Option** | **Number of CCGs (n)** | **Percentage (%) (n=129)** |
| --- | --- | --- |
| I have | 69 | 53% |
| CCG prescribing advisor | 67 | 52% |
| CCG practice pharmacist | 37 | 29% |
| NHSE GP practice pharmacist | 2 | 2% |
| Local microbiologist | 76 | 59% |
| NHSE GP practice antimicrobial lead/champion | 20 | 16% |
| Other | 37 | 29% |
| **Total that reported who had delivered education/training** | **129** |  |
| Don't know | 1 |  |
| **Total that completed question** | **130** |  |
| Blank | 10 |  |

## Over the last 2 years (2015-2017), what resources have you/they use in this AMS education/training? (n=140. Those who indicated that their primary care practitioners had received face-to-face AMS education/training in the last two years (2015-2017))

| **Option** | **Number of CCGs (n)** | **Percentage (%) (n=132)** |
| --- | --- | --- |
| Locally developed AMS education/training | 83 | 63% |
| TARGET antibiotic group presentation | 70 | 53% |
| Detailed action planning | 21 | 16% |
| Antimicrobial prescribing data | 117 | 89% |
| Audit tools | 70 | 53% |
| Other | 17 | 13% |
| **Total that reported what resources have been used in their AMS education/training** | **132** |  |
| Don't know | 1 |  |
| **Total that completed question** | **133** |  |
| Blank | 7 |  |

## Over the last 2 years (2015-17), approximately, what percentage of primary care practices in your CCG(s) have received face-to-face AMS education/training? (n=140. Those who indicated that their primary care practitioners had received face-to-face AMS education/training in the last two years (2015-2017))

| **Option** | **Number of CCGs (n)** | **Percentage (%) (n=106)** |
| --- | --- | --- |
| <25% | 9 | 8% |
| 25-50% | 4 | 4% |
| 51-75% | 20 | 19% |
| >75% | 73 | 69% |
| **Total that reported what percentage of primary care practices in their CCG(s) had received face-to-face AMS education/training** | **106** |  |
| Don't know | 22 |  |
| **Total that completed question** | **128** |  |
| Blank | 12 |  |

## Over the last 2 years (2015-2017), which e-learning have you signposted your primary care practitioners to? (n=121. Those who indicated that their primary care practitioners had been signposted to AMS e-learning in the last two years (2015-2017))

| **Option** | **Number of CCGs (n)** | **Percentage (%) (n=119)** |
| --- | --- | --- |
| BSAC’s MOOC (Massive Open Online Course) | 10 | 8% |
| HEE (Health Education England) ‘Reducing Antimicrobial Resistance: An Introduction’ | 33 | 28% |
| NICE AMS course | 20 | 17% |
| e-LfH (eLearning for Healthcare) Level 1 AMR | 33 | 28% |
| TARGET eLearning resources eg. TARGET antibiotic webinar series, TARGET Antibiotic Resistance in Primary Care e-module, Skin Infections online course, MARTI Managing Acute Respiratory Tract Infections, Urinary Tract Infections, Managing infectious diarrhoea, Sexual Health in Primary Care | 117 | 98% |
| CPPE (Centre for Pharmacy Postgraduate Education) resources | 18 | 15% |
| Other | 8 | 7% |
| **Total that reported which e-learning their primary care practitioners have been signposted to** | **119** |  |
| Blank | 2 |  |

## Over the last 2 years (2015-2017), which TARGET Antibiotics toolkit e-Learning resources have you signposted your primary care practitioners to? (n=117. Those that indicated that their primary care practitioners had been signposted to ‘TARGET eLearning resources eg. TARGET antibiotic webinar series, TARGET Antibiotic Resistance in Primary Care e-module, Skin Infections online course, MARTI Managing Acute Respiratory Tract Infections, Urinary Tract Infections, Managing infectious diarrhoea, Sexual Health in Primary Care’)

| **Option** | **Number of CCGs (n)** | **Percentage (%) (n=103)** |
| --- | --- | --- |
| TARGET antibiotic webinar series | 77 | 75% |
| TARGET Antibiotic Resistance in Primary Care e-module | 73 | 71% |
| Skin Infections online course | 19 | 18% |
| MARTI Managing Acute Respiratory Tract Infections | 31 | 30% |
| Urinary Tract Infections | 47 | 46% |
| Managing infectious diarrhoea | 11 | 11% |
| Sexual Health in Primary Care | 13 | 13% |
| STAR: Stemming the Tide of Antibiotic Resistance e-module | 20 | 19% |
| National Prescribing Centre (NPC) e-learning | 9 | 9% |
| **Total that reported which TARGET e-learning their primary care practitioners have been signposted to** | **103** |  |
| Don’t know | 10 |  |
| **Total that completed question** | **113** |  |
| Blank | 4 |  |

## Over the last 2 years (2015-2017), have you monitored how many primary care practitioners have completed the e-learning you signpost to? (n=121. Those who indicated that their primary care practitioners had been signposted to AMS e-learning in the last two years (2015-2017))

| **Option** | **Number of CCGs (n)** | **Percentage (%) (n=117)** |
| --- | --- | --- |
| No | 100 | 85% |
| Yes | 13 | 11% |
| Don't know | 4 | 3% |
| **Total that completed question** | **117** |  |
| Blank | 4 |  |

## Over the last 2 years (2015-2017), have you, yourself, accessed/received any education/training on AMS in your role? (n=187)

| **Option** | **Number of CCGs (n)** | **Percentage (%) (n=169)** |
| --- | --- | --- |
| No | 49 | 29% |
| Yes | 118 | 70% |
| Don't know | 2 | 1% |
| **Total that completed question** | **169** |  |
| Blank | 18 |  |

## To what extent do you think education/training, generally, is successful in supporting antimicrobial stewardship in primary care? (n=187)

| **Option** | **Number of CCGs (n)** | **Percentage (%) (n=134)** |
| --- | --- | --- |
| Very UNsuccessful | 1 | 1% |
| UNsuccessful | 0 | 0% |
| Neutral | 18 | 13% |
| Successful | 81 | 60% |
| Very Successful | 34 | 25% |
| **Total that evaluated** | **134** |  |
| We DO promote/access this, but I don't know how successful it is | 31 |  |
| Don't know as we DO NOT currently promote/access this | 3 |  |
| **Total that completed question** | **168** |  |
| Blank | 19 |  |

## How important are the following characteristics of education/training in relation to facilitators for AMS in your CCG(s)? (n=187)

| **Option** | **Face-to-face education/training for primary care practitioner (number of responses/164 (%))** | **Education/training for primary care practitioners delivered by a local/respected expert in the field ie. Antimicrobial pharmacist, microbiologist etc (number of responses/166 (%))** | **CCG/CSUs to be able to request education/training from a reputable source eg. PHE (number of responses/168 (%))** | **Mixing up the format of education/training so it seems less repetitive to primary care practitioner (number of responses/167 (%))** | **Education/training including real-life relatable clinical case studies (number of responses/167 (%))** | **Education/training that emphasises the link between appropriate antimicrobial prescribing and reducing workload for the practitioner (number of responses/165 (%))** | **Primary care-specific e-learning (number of responses/168 (%))** | **Education/training for the whole practice (number of responses/166 (%))** | **Tailored education/training for antimicrobial prescribers (number of responses/165 (%))** | **Making AMS education/training mandatory for all primary care practitioner (number of responses/166 (%))** |
| --- | --- | --- | --- | --- | --- | --- | --- | --- | --- | --- |
| Very Unimportant | 0 (0%) | 0 (0%) | 1 (1%) | 0 (0%) | 0 (0%) | 1 (1%) | 0 (0%) | 0 (0%) | 0 (0%) | 1 (1%) |
| Unimportant | 1 (1%) | 0 (0%) | 6 (4%) | 0 (0%) | 3 (2%) | 0 (0%) | 2 (1%) | 5 (3%) | 5 (3%) | 0 (0%) |
| Neutral | 16 (10%) | 12 (7%) | 24 (14%) | 28 (17%) | 16 (10%) | 16 (10%) | 48 (29%) | 31 (19%) | 35 (21%) | 35 (21%) |
| Important | 94 (57%) | 76 (46%) | 91 (54%) | 81 (49%) | 76 (46%) | 76 (46%) | 87 (52%) | 101 (61%) | 91 (55%) | 80 (48%) |
| Very important | 53 (32%) | 78 (47%) | 46 (27%) | 58 (35%) | 72 (43%) | 72 (44%) | 31 (18%) | 29 (17%) | 34 (21%) | 50 (30%) |
| **Total that completed question** | **164** | **166** | **168** | **167** | **167** | **165** | **168** | **166** | **165** | **166** |
| Blank | 23 | 21 | 19 | 20 | 20 | 22 | 19 | 21 | 22 | 21 |

# TARGET

## Over the last 2 years (2015-2017), have you actively promoted use of the TARGET Antibiotics Toolkit (available on the RCGP website) to support antimicrobial stewardship in primary care? (n=187)

| **Option** | **Number of CCGs (n)** | **Percentage (%) (n=186)** |
| --- | --- | --- |
| No, we do NOT actively promote the TARGET antibiotics Toolkit | 2 | 1% |
| Yes, we actively promote the TARGET Antibiotics Toolkit | 184 | 99% |
| I've never heard of the TARGET Antibiotics Toolkit | 0 | 0% |
| Don't know | 0 |  |
| **Total that completed question** | **186** |  |
| Blank | 1 |  |

## Over the last 2 years (2015-2017), which resources from the TARGET Antibiotics Toolkit have you actively promoted for your primary care practitioners to use? (n=187)

| **Option** | **Number of CCGs (n)** | **Percentage (%) (n=175)** |
| --- | --- | --- |
| TYI-RTI leaflet | 161 | 92% |
| Get well soon without antibiotics leaflet | 138 | 79% |
| TYI-UTI leaflet | 117 | 67% |
| Antibiotic Guardian leaflet | 117 | 67% |
| When should I worry? booklet for parents and carers | 113 | 65% |
| Managing your Infection leaflet | 61 | 35% |
| Caring for children with coughs leaflet | 47 | 27% |
| **Total that reported which TARGET tools were actively promoted** | **175** |  |
| None | 4 |  |
| Don't know | 7 |  |
| **Total that completed question** | **186** |  |
| Blank | 1 |  |

## What formats have you provided these leaflets in for your primary care practitioners? (n=182. Not including the 4 who indicated ‘None’ for promoting leaflets and the 1 that left blank)

| **Option** | **Print and give hard copy to GP staff (n (% of total that reported format of leaflet provision))** | **Signpost to the TARGET website (n (% of total that reported promoting this leaflet))** | **Integrated into clinical system (n (% of total that reported promoting this leaflet))** | **Added to local primary care antimicrobial guidance/formulary (n (% of total that reported promoting this leaflet))** | **Total that reported format of leaflet provision** | **Don't know** | **NA** | **Blank** |
| --- | --- | --- | --- | --- | --- | --- | --- | --- |
| Get well soon without antibiotics leaflet (n=138) | 82 (63%) | 94 (72%) | 10 (8%) | 23 (18%) | **131** | 0 | 1 | 6 |
| Caring for children with coughs leaflet (n=47) | 13 (28%) | 41 (89%) | 4 (9%) | 6 (13%) | **46** | 0 | 0 | 1 |
| When should I worry? booklet for parents and carers (n=113) | 53 (50%) | 90 (84%) | 6 (6%) | 25 (23%) | **107** | 0 | 1 | 5 |
| Managing your Infection leaflet (n=61) | 23 (39%) | 49 (83%) | 6 (10%) | 6 (10%) | **59** | 0 | 0 | 2 |
| Antibiotic Guardian leaflet (n=117) | 63 (55%) | 88 (77%) | 8 (7%) | 16 (14%) | **115** | 0 | 0 | 2 |
| TYI-RTI leaflet (n=161) | 83 (53%) | 123 (78%) | 65 (41%) | 61 (39%) | **157** | 0 | 0 | 4 |
| TYI-UTI leaflet (n=117) | 46 (40%) | 97 (84%) | 28 (24%) | 45 (39%) | **115** | 0 | 1 | 1 |

## To which practitioners have you actively promoted these leaflets to? (n=182. Not including the 4 who indicated ‘None’ for promoting leaflets and the 1 that left blank)

| **Option** | **GP staff (n (% of total number of CCGs that reported to which practitioners the leaflet had been promoted to))** | **School nurses (n (% of total number of CCGs that reported to which practitioners the leaflet had been promoted to))** | **Community pharmacists (n (% of total number of CCGs that reported to which practitioners the leaflet had been promoted to))** | **Out of Hours staff (n (% of total number of CCGs that reported to which practitioners the leaflet had been promoted to))** | **Total number of CCGs that reported to which practitioners the leaflet had been promoted to** | **Don't know** | **NA** | **Blank** |
| --- | --- | --- | --- | --- | --- | --- | --- | --- |
| Get well soon without antibiotics leaflet (n=138) | 132 (100%) | 1 (1%) | 72 (55%) | 35 (27%) | **132** | 0 | 0 | 6 |
| Caring for children with coughs leaflet (n=47) | 42 (98%) | 3 (7%) | 15 (35%) | 12 (28%) | **43** | 3 | 0 | 1 |
| When should I worry? booklet for parents and carers (n=113) | 102 (100%) | 6 (6%) | 32 (31%) | 26 (25%) | **102** | 7 | 0 | 4 |
| Managing your Infection leaflet (n=61) | 55 (96%) | 1 (2%) | 22 (39%) | 14 (25%) | **57** | 0 | 0 | 4 |
| Antibiotic Guardian leaflet (n=117) | 107 (99%) | 10 (9%) | 52 (48%) | 36 (33%) | **108** | 0 | 0 | 9 |
| TYI-RTI leaflet (n=161) | 158 (100%) | 5 (3%) | 61 (39%) | 50 (32%) | **158** | 0 | 0 | 3 |
| TYI-UTI leaflet (n=117) | 112 (100%) | 4 (4%) | 29 (26%) | 32 (29%) | **112** | 0 | 0 | 5 |

## Currently, how is the TARGET Treating Your Infection (TYI) leaflet promoted in your local primary care antimicrobial guidelines? (n=182. Not including the 4 who indicated ‘None’ for promoting leaflets and the 1 that left blank)

| **Option** | **Number of CCGs (n)** | **Percentage (%) (n=128)** |
| --- | --- | --- |
| To support back-up/delayed prescribing | 107 | 84% |
| To support self-care and safety netting | 100 | 78% |
| To support appropriate prescribing | 114 | 89% |
| Other | 22 | 17% |
| **Total that reported promoting the TYI leaflet in their local primary care antimicrobial guidelines** | **128** |  |
| The TYI leaflet is NOT currently promoted in our primary care antimicrobial guidelines | 45 |  |
| Don't know | 7 |  |
| **Total that completed question** | **180** |  |
| Blank | 2 |  |

## To what extent do you think the TARGET Antibiotics Toolkit leaflets to share with patients are successful in supporting antimicrobial stewardship in primary care? (n=187)

| **Option** | **Very UNsuccessful (n (% of total number of CCGs that evaluated))** | **UNsuccessful (n (% of total number of CCGs that evaluated))** | **Neutral (n (% of total number of CCGs that evaluated))** | **Successful (n (% of total number of CCGs that evaluated))** | **Very successful (n (% of total number of CCGs that evaluated))** | **Total number of CCGs that reported success** | **We DO promote this resource, but I don’t know how successful it is** | **Don’t know as we DO NOT currently promote this resource** | **Blank** |
| --- | --- | --- | --- | --- | --- | --- | --- | --- | --- |
| TYI-UTI leaflet | 0 (0%) | 2 (6%) | 12 (38%) | 17 (53%) | 1 (3%) | **32** | 98 | 30 | 27 |
| TYI-RTI leaflet | 0 (0%) | 0 (0%) | 11 (13%) | 56 (68%) | 15 (18%) | **82** | 87 | 8 | 10 |
| TYI-RTI (Pictorial leaflet) | 0 (0%) | 1 (4%) | 7 (26%) | 17 (63%) | 2 (7%) | **27** | 52 | 60 | 48 |
| Antibiotic Guardian leaflet | 0 (0%) | 2 (4%) | 23 (47%) | 21 (43%) | 3 (6%) | **49** | 77 | 32 | 29 |
| When should I worry? booklet for parents and carers | 0 (0%) | 2 (6%) | 14 (40%) | 16 (46%) | 3 (9%) | **35** | 82 | 30 | 40 |
| Caring for children with coughs leaflet | 0 (0%) | 1 (4%) | 13 (54%) | 10 (42%) | 0 (0%) | **24** | 41 | 68 | 54 |
| Get well soon without antibiotics leaflet | 0 (0%) | 2 (3%) | 19 (33%) | 34 (59%) | 3 (5%) | **58** | 66 | 32 | 31 |
| Leaflets to share with patients (overall) | 0 (0%) | 1 (2%) | 18 (29%) | 38 (61%) | 5 (8%) | **62** | 61 | 22 | 42 |

## Over the last 2 years (2015-2017), which other resources from the TARGET Antibiotics Toolkit have you actively promoted for your primary care practitioners to use? (n=187)

| **Option** | **Number of CCGs (n)** | **Percentage (%) (n=146)** |
| --- | --- | --- |
| Videos for patient waiting areas | 84 | 58% |
| Self care forum fact sheets | 55 | 38% |
| Posters | 137 | 94% |
| **Total that reported actively promoting the TARGET resources for clinical and waiting areas** | **146** |  |
| Don't know | 21 |  |
| None | 19 |  |
| **Total that completed question** | **186** |  |
| Blank | 1 |  |

## To what extent do you think the TARGET Antibiotics Toolkit resources for clinical and waiting areas are successful in supporting antimicrobial stewardship in primary care? (n=187)

| **Option** | **Very UNsuccessful (n (% of total number of CCGs that reported success))** | **UNsuccessful (n (% of total number of CCGs that reported success))** | **Neutral (n (% of total number of CCGs that reported success))** | **Successful (n (% of total number of CCGs that reported success))** | **Very successful (n (% of total number of CCGs that reported success))** | **Total number of CCGs that reported success** | **We DO promote this resource, but I don’t know how successful it is** | **Don’t know as we DO NOT currently promote this resource** | **Blank** |
| --- | --- | --- | --- | --- | --- | --- | --- | --- | --- |
| Posters | 0 (0%) | 1 (2%) | 14 (21%) | 44 (67%) | 7 (11%) | **66** | 81 | 23 | 17 |
| Self care forum fact sheets | 0 (0%) | 0 (0%) | 11 (55%) | 5 (25%) | 4 (20%) | **20** | 50 | 77 | 40 |
| Videos for patient waiting areas | 0 (0%) | 4 (10%) | 11 (26%) | 18 (43%) | 9 (21%) | **42** | 61 | 60 | 24 |
| Resources for clinical and waiting areas (overall) | 0 (0%) | 0 (0%) | 4 (8%) | 42 (81%) | 6 (12%) | **52** | 53 | 43 | 39 |

## Over the last 2 years (2015-2017), have you actively promoted use of the TARGET Antibiotics Toolkit self-assessment checklist for primary care prescribers? (n=187)

| **Option** | **Number of CCGs (n)** | **Percentage (%) (n=180)** |
| --- | --- | --- |
| Yes | 98 | 54% |
| No | 66 | 9% |
| Don't know | 16 | 37% |
| **Total that completed question** | **180** |  |
| Blank | 7 |  |

## To what extent do you think the TARGET Antibiotics Toolkit self-assessment checklist for primary care prescribers is successful in supporting antimicrobial stewardship in primary care? (n=187)

| **Option** | **Number of CCGs (n)** | **Percentage (%) (n=45)** |
| --- | --- | --- |
| Very Unsuccessful | 0 | 0% |
| Unsuccessful | 4 | 9% |
| Neutral | 21 | 47% |
| Successful | 13 | 29% |
| Very successful | 7 | 16% |
| **Total that reported success** | **45** |  |
| We DO promote this resource, but I don’t know how successful it is | 65 |  |
| Don't know as we DO NOT currently promote this resource | 64 |  |
| **Total that completed question** | **174** |  |
| Blank | 13 |  |

## Approximately what percentage of primary care practitioners use the TARGET Antibiotic Toolkit resources? (n=187)

| **Option** | **Number of CCGs (n)** | **Percentage of total number of CCGs (n=39)** |
| --- | --- | --- |
| <5% | 3 | 8% |
| 25% | 7 | 18% |
| 50% | 7 | 18% |
| 75% | 15 | 38% |
| >90% | 7 | 18% |
| **Total that reported which percentage of primary care practitioners use the TARGET resources** | **39** |  |
| Don't know | 137 |  |
| **Total that completed question** | **176** |  |
| Blank | 11 |  |

## As a commissioner, over the last 2 years (2015-2017), which resources from the TARGET Antibiotics toolkit have you used yourself? (n=187)

| **Option** | **Number of CCGs (n)** | **Percentage (%) (n=133)** |
| --- | --- | --- |
| Guide to resources | 100 | 75% |
| Self assessment checklist for commissioners | 60 | 45% |
| Trainers and commissioners background presentation | 58 | 44% |
| **Total that reported personally using the TARGET toolkit in the last 2 years (2015-2017)** | **133** |  |
| None | 39 |  |
| **Total that completed question** | **172** |  |
| Blank | 15 |  |

## Over the last 2 years (2015-2017), have you used any other tools to make any assessment of current antimicrobial stewardship initiatives across your CCG(s)? (n=187)

| **Option** | **Number of CCGs (n)** | **Percentage (%) (n=104)** |
| --- | --- | --- |
| Yes, we use the NICE baseline assessment tool | 78 | 75% |
| Yes, we use 'other' tools to assess our AMS initiatives across our CCG(s) | 40 | 38% |
| **Total that reported that they had used other tools to assess current antimicrobial stewardship initiatives** | **104** |  |
| No, we don’t use anything else to assess our AMS initiatives across our CCG(s) | 47 |  |
| Don’t know | 18 |  |
| **Total that completed question** | **169** |  |
| Blank | 18 |  |

## When was the last time you used these resources for commissioners? (n=133. Not including the 39 that reported that they had not accessed the resources for commissioners, or the 15 that left blank)

| **Option** | **TARGET Guide to resources (number of responses/102 (%))** | **TARGET Trainers and commissioners background presentation (number of responses/67 (%))** | **TARGET Self assessment checklist for commissioners (number of responses/64 (%))** | **NICE baseline assessment tool (number of responses/76 (%))** | **'Other' tools to assess current AMS activity (number of responses/41 (%))** |
| --- | --- | --- | --- | --- | --- |
| In the last 6 months | 36 (35%) | 11 (16%) | 16 (25%) | 17 (22%) | 29 (71%) |
| In the last year | 35 (34%) | 19 (28%) | 19 (30%) | 31 (41%) | 6 (15%) |
| In the last 1-2 years | 25 (25%) | 23 (34%) | 21 (33%) | 19 (25%) | 3 (7%) |
| Over 2 years ago | 6 (6%) | 14 (21%) | 8 (13%) | 9 (12%) | 3 (7%) |
| **Total that reported when they had last accessed the TARGET resource** | **102** | **67** | **64** | **76** | **41** |
| NA | 19 | 41 | 45 | 37 | 57 |
| **Total that completed question** | **121** | **108** | **109** | **113** | **98** |
| Blank | 12 | 25 | 24 | 20 | 35 |

## To what extent do you think the TARGET Antibiotics self-assessment checklist for commissioners is successful in supporting antimicrobial stewardship in primary care? (n=60. Those that indicated that they had used the Self assessment checklist for commissioners in the last two years (2015-2017))

| **Option** | **Number of CCGs (n)** | **Percentage (%) (n=48)** |
| --- | --- | --- |
| Very Unsuccessful | 0 | 0% |
| Unsuccessful | 3 | 6% |
| Neutral | 21 | 44% |
| Successful | 22 | 46% |
| Very successful | 2 | 4% |
| **Total that reported success** | **48** |  |
| Don't know | 10 |  |
| **Total that completed question** | **58** |  |
| Blank | 2 |  |

# Antibiotic Guardian Campaign

## Over the last 2 years (2015-2017), how have you actively promoted the antibiotic guardian campaign? (n=187)

| **Option** | **Number of CCGs (n)** | **Percentage (%) (n=132)** |
| --- | --- | --- |
| Encourage primary care practitioners to become antibiotic guardians | 129 | 98% |
| Encourage patients and the public to become antibiotic guardians | 74 | 56% |
| Other | 6 | 5% |
| **Total that actively encourage others to become antibiotic guardians** | **132** |  |
| I've never heard of the Antibiotic Guardian Campaign | 0 |  |
| We do NOT actively encourage people to become antibiotic guardians | 30 |  |
| Don't know | 5 |  |
| **Total that completed question** | **167** |  |
| Blank | 20 |  |

## To what extent do you think the antibiotic guardian campaign is successful at raising awareness and prioritising AMS activity for your primary care practitioners? (n=167. Not including the 20 that left blank)

| **Option** | **Success of Antibiotic Guardian (Awareness of AMS) (number of responses/96 (%))** | **Success of Antibiotic Guardian (Prioritising AMS) (number of responses/95 (%))** |
| --- | --- | --- |
| Very Unsuccessful | 2 (2%) | 2 (2%) |
| Unsuccessful | 9 (9%) | 10 (11%) |
| Neutral | 30 (31%) | 47 (49%) |
| Successful | 51 (53%) | 32 (34%) |
| Very successful | 4 (4%) | 4 (4%) |
| **Total that reported success** | **96** | **95** |
| We DO promote this campaign, but I don't know how successful it is | 36 | 38 |
| Don't know as we DO NOT currently promote this campaign | 27 | 27 |
| **Total that completed question** | **159** | **160** |
| Blank | 8 | 7 |
